# Interplay between flaviviruses and the interferon response: mechanisms of immune evasion and therapeutic implications

**DOI:** 10.3389/fphar.2026.1745652

**Published:** 2026-02-18

**Authors:** Jiayin Han, Yue Yin, Jing Yuan

**Affiliations:** 1 Department of Infectious Diseases, The Affiliated Hospital of Southwest Medical University, Luzhou, China; 2 Shenzhen Key Laboratory of Pathogen and Immunity, State Key Discipline of Infectious Disease, Shenzhen Third People’s Hospital, Second Hospital Affiliated with Southern University of Science and Technology, Shenzhen, China

**Keywords:** orthoflavivirus, type I interferon, type III interferon, innate immunity, viral antagonism, blood-brain barrier, placental barrier

## Abstract

The genus Orthoflavivirus encompasses a group of medically significant arthropod-borne viruses, such as dengue virus (DENV), Japanese encephalitis virus West Nile virus Zika virus (ZIKV), and others, which pose persistent global public health threats. The host interferon (IFN) system constitutes a pivotal first line of defense against these viral infections. However, orthoflaviviruses have evolved a remarkable array of sophisticated strategies to antagonize both the induction and signaling pathways of type I and III IFNs. This review systematically summarizes the mechanisms by which orthoflaviviruses evade the IFN response, primarily by employing viral proteins to target key host factors in pattern recognition receptor signaling pathways (e.g., RIG-I, MDA5, MAVS, TBK1, STING, IRF3) and the JAK-STAT signaling cascade (e.g., STAT1, STAT2, IFNAR1). Furthermore, we highlight the critical and complex roles of IFNs at the placental and blood-brain barriers (BBB), the primary sites for transplacental transmission and neuroinvasion. At these barriers, IFNs play a dual role: they exert essential antiviral effects to restrict viral replication, but may also contribute to immunopathology. The delicate balance of IFN responses determines the outcomes of congenital infection and neurological diseases. Finally, we discuss the therapeutic potential of harnessing the IFN pathway, while also acknowledging the challenges and need for further *in vivo* studies to translate these insights into effective treatments for flavivirus-associated diseases.

## 1 Introduction

When a virus invades our bodies, it triggers an immediate and complex battle within. Leading the charge is our innate immune system-a rapid-response defense network that works to contain and eliminate threats. -Among its most critical weapons are interferons (IFNs), a family of signaling proteins renowned for their potent antiviral power. While type I and II IFNs have long been studied for their broad protective roles, recent research has highlighted the specialized importance of type III IFNs, particularly in safeguarding vulnerable sites such as our mucosal surfaces and the placenta during pregnancy (Wells and Coyne, 2018; Walker et al., 2021; Doldan et al., 2022).

Orthoflavivirus is a genus of enveloped, positive-sense single-stranded RNA viruses transmitted primarily by mosquitoes, encompassing more than 70 species. While only a subset of these infect humans, they contribute significantly to global morbidity and mortality. Japanese encephalitis virus (JEV), endemic mainly across Asia, cause approximately 67,900 reported cases annually and can be fatal in severe forms (Zheng et al., 2012). West Nile virus (WNV), first isolated in Uganda, leads to neuroinvasive disease-such as encephalitis or acute flaccid paralysis-in about 1% of infections and is associated with considerable mortality (Smithburn et al., 1940; Davis et al., 2006). Dengue virus (DENV) accounts for an estimated 100–400 million new infections each year, with a proportion progressing to life-threatening dengue hemorrhagic fever (DHF) or dengue shock syndrome (DSS) (Kularatne and Dalugama, 2022; Murphy and Whitehead, 2011). Yellow fever virus (YFV) remains entrenched in regions of Africa and the Americas, typically manifesting as a hemorrhagic fever with jaundice (Garske et al., 2014). Zika virus (ZIKV), though often mild, can cross the placental and blood-brain barriers, resulting in congenital microcephaly and Guillain-Barré syndrome in adults, thereby posing distinct risks during pregnancy and to the nervous system. These five viruses exemplify the three principal severe disease patterns associated with flaviviruses: neurotropism (JEV, WNV), hemorrhagic fever (DENV, YFV), and congenital injury (ZIKV). Given their demonstrated potential for transnational spread or re-emergence, they form the central focus of this review.

The interplay between IFNs and viruses is a dynamic evolutionary arms race. On one side, viruses have developed sophisticated tactics to evade or neutralize interferon-mediated defenses-often by producing proteins that block IFN production, signaling, or function. On the other, our immune system continually adapts to strengthen its antiviral arsenal. The outcome of each infection largely depends on this delicate balance between viral evasion and host defense (Zhu and Zheng, 2020; Yin and Favoreel, 2021).

This review aims to provide a comprehensive overview of the current understanding of the mechanisms by which flaviviruses evade or counteract the antiviral effects of IFNs. Furthermore, we highlight the role of type I and type III interferons in the blood-brain barrier and placental barrier during ZIKV infection. Finally, we discuss the potential therapeutic implications of targeting Type I and Type III interferon signalling pathways for the prevention and treatment of flavivirus-associated diseases. By synthesizing the existing knowledge in this field, we hope to provide insights into complex host-virus interactions and identify new avenues for future research.

## 2 Orthoflaviviruses

The genus *Orthoflaviviruses* belongs to the Flaviviridae family, which contains a variety of human pathogens, the majority of which are arthropod-borne, such as JEV, WNV, DENV, YFV, and ZIKV, which have caused severe epidemics in the past and have the potential to present significant threats to public health in the future (Postler et al., 2023).

**FIGURE 1:**
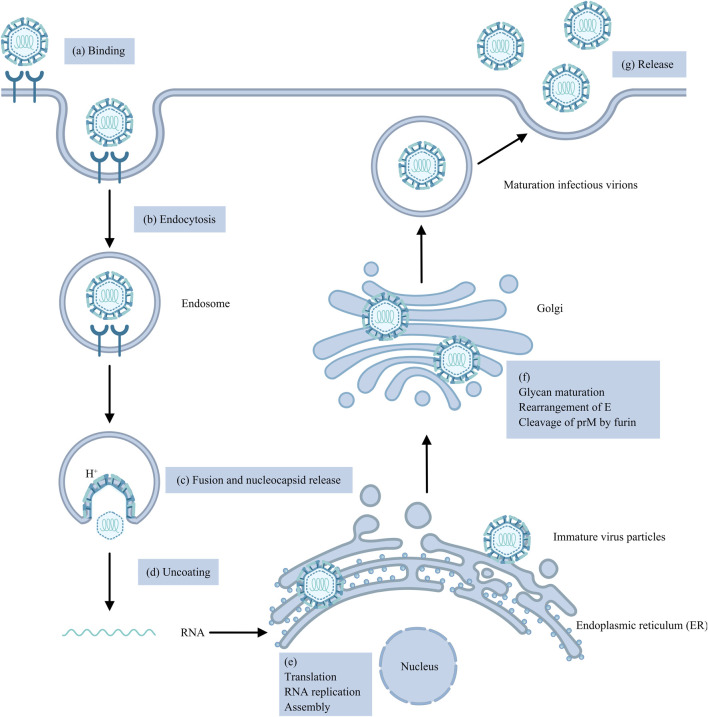
The Orthoflavivirus replication cycle. **(a)** The virus binds to the cell surface. **(b)** The virus enters the cell primarily through clathrin-mediated endocytosis. **(c)** Low-pH endosomes lead to the fusion of the viral E protein with the endosomal membrane and the release of the NC. **(d)** Viral RNA uncoating. **(e)** Genomic RNA is translated into a single polyprotein, which is then cleaved by NS2B-NS3 proteases and host signal peptidases; RNA replication involves the synthesis of viral RNA and capping of nascent positive-strand RNA; and viral RNA and C proteins form an NC and then envelop into immature virus particles. **(f)** Immature virus particles mature into infectious virions through glycan maturation in the Golgi apparatus, rearrangement of E proteins and cleavage of prM. **(g)** Mature infectious virions are released from cells by exocytosis.

Flaviviruses are enveloped viruses that include a +ssRNA genome encoding a single open reading frame (ORF) that can be translated into a polyprotein that is cleaved into three structural proteins, capsid (C), precursor membrane (prM) and envelope (E), and seven non-structural proteins, NS1, NS2A, NS2B, NS3, NS4A, NS4B and NS5, by viral and host proteases (Pierson and Diamond, 2020; Fisher et al., 2023). The first step of the virus life cycle involves binding to the cell surface, after which the virus enters the host cell mainly through clathrin-mediated endocytosis (Hu et al., 2021). The acidic environment of the endosome induces conformational changes and rearrangements of the viral E protein, leading to the fusion of the viral E protein and endosomal membrane, which results in the release of the nucleocapsid (NC) into the cytoplasm (Hu et al., 2021; Knyazhanskaya et al., 2021; Pan et al., 2022). Following the uncoating of the viral RNA, which requires the dissociation of the C protein and viral RNA, the +ssRNA is directly translated into a polyprotein on the rough endoplasmic reticulum (ER), which is subsequently cleaved by NS2B-NS3 proteases and host signal peptidases (Van Den Elsen et al., 2021; Jablunovsky and Jose, 2024). Both viral genome RNA replication and virus particle assembly occur in the ER. Replication includes the synthesis of viral RNA and the capping of nascent positive-strand RNA (Knyazhanskaya et al., 2021). NS proteins, along with viral RNA, host proteins and a series of host factors, form membrane-bound replication complexes (RCs) in ER-derived vesicle packets (VPs), where viral RNA is synthesized (Knyazhanskaya et al., 2021; Selisko et al., 2014; Roby et al., 2015). During the primary stage of genome replication, positive-strand RNA acts as a template for the synthesis of complementary negative-strand RNA under the influence of NS5 RNA-dependent RNA polymerase (RdRp), thereby giving rise to a double-stranded RNA (dsRNA) replication intermediate (Tan et al., 2023). The negative-strand RNA of the dsRNA replication intermediate then acts as a template for the synthesis of the positive-strand RNA by NS5 RNA polymerase. Moreover, the new positive-strand RNA replaces the original RNA in the dsRNA replication intermediate. The nascent dsRNA replication intermediate serves as a template to generate more copies of positive-strand RNA (Knyazhanskaya et al., 2021). The replication of genomic RNA is asymmetrically semiconserved (Roby et al., 2015). The new positive-strand RNA is subsequently 5′-capped and methylated by the NS3 helicase and NS5 MTase domain. Viral RNA and the C protein constitute a nucleocapsid that is enveloped by prM, E proteins, and the cellular lipid bilayer, giving rise to immature virus particles that bud into the ER lumen (Knyazhanskaya et al., 2021; Jablunovsky and Jose, 2024; Morita and Suzuki, 2021). Next, immature virus particles undergo a series of processes, such as glycan maturation in the Golgi apparatus, rearrangement of E proteins and cleavage of prM into Pr and M proteins by furin proteases in the trans-Golgi network (TGN), creating mature infectious virions that are released from the host cell by exocytosis (Roby et al., 2015). In brief, the life cycle of Genus Orthoflaviviruses is complicated and regulated by multifunctional structural and non-structural proteins (Figure 1).

### 2.1 Japanese encephalitis virus

Japanese encephalitis (JE) is the most important virus encephalitis worldwide, with nearly 67,900 JE cases occurring in 24 JE-endemic Asian and Western Pacific countries each year, approximately half of which occur in China (excluding Taiwan), and approximately 75% occur in children (0–14 years old) (Zheng et al., 2012; Campbell et al., 2011). While the majority of JEV infections are asymptomatic, a small proportion progress to encephalitis, of which 20%–30% are fatal. The transmission cycle of JEV involves mosquitoes, vertebrate hosts and environmental factors (Chugh et al., 2025). *Culex mosquitoes*, primarily *Culex tritaeniorhynchus*, serve as vectors, transmitting the virus between birds-such as egrets and herons, which act as natural reservoirs-and pigs, which function as amplifying hosts (Mulvey et al., 2021). Humans and horses are considered dead-end hosts, as they do not develop sufficient viremia to sustain onward transmission (Chugh et al., 2025). Furthermore, climate change, intensified agricultural practices, and environmental modificationshave contributed to the continued geographical expansion of JEV (Chugh et al., 2025). For instance, in March 2022, an outbreak was reported in temperate southeastern Australia, whereas previously JEV had been documented only in localized outbreaks in the tropical northeastern part of the country (Reyes et al., 2025).

### 2.2 West nile virus

West Nile virus, a member of the Japanese encephalitis serocomplex (JES), which was first isolated from the blood of an African febrile woman in 1937, is currently among the most widespread vector-borne flaviviruses in the world (Smithburn et al., 1940; Petersen et al., 2013; Giesen et al., 2023). Approximately 80% of patients with WNV infection are asymptomatic, 20% develop West Nile fever (WNF), and less than 1% develop West Nile neuroinvasive disease (WNND), including syndromes of meningitis, encephalitis, and acute flaccid paralysis (AFP)/poliomyelitis (Davis et al., 2006). The WNV is maintained in nature through a transmission cycle involving avian hosts and ornithophilic mosquito vectors (Martín-Acebes and Saiz, 2012). Humans and horses are considered accidental, dead-end hosts, as they typically do not develop sufficient viremia to sustain viral transmission, and primary WNV vectors predominantly feed on birds (Saiz et al., 2021). Therefore, reducing mosquito density and preventing human exposure to mosquito bites remain crucial strategies for interrupting WNV transmission (Singh et al., 2025). Nevretheless, accumulating evidence indicates that rising temperatures have enhanced mosquito vector activity, thus expanding the transmission range of WNV (Singh et al., 2025).

### 2.3 Dengue virus

DENVs include four serotypes, DENV-1, DENV-2, DENV-3 and DENV-4, all of which can induce dengue, which is highly prevalent in tropical and subtropical climates, with an estimated 100–400 million new infections annually (Kularatne and Dalugama, 2022). The clinical manifestations of dengue range widely from symptomless infection to dengue fever (DF), DHF and DSS, the latter two of which are more severe and potentially life-threatening (Murphy and Whitehead, 2011). Unlike JEV and WNV, DENV infection in humans produces high-level viremia that allows the virus to pass efficiently between mosquito vectors and people, forming a “human-mosquito-human” transmission loop that sustains its continuous prevalence (Whitehead et al., 2007). In this cycle, *Aedes aegypti* plays the main role, while *Aedes albopictus* serves as the auxiliary vector.

Moreover, numerous studies have indicated that a distinct seasonal pattern in DENV outbreaks. In Guangdong, China’s most dengue-affected province, outbreaks consistently peak between July and October each year (Cui et al., 2022). Similarly, in Bangladesh, transmission intensifies from June to August (Hasan et al., 2024). These seasonal surges are largely precipitation-driven, as heavy rainfall creates extensive mosquito breeding sites and accelerates DENV transmission.

The first recorded outbreak of DENV can be traced back to 1779, after which recurrent epidemics have occurred across the Americas, Africa, and Asia (Roy and Bhattacharjee, 2021). In 2024, DENV continued its global expansion, with over 14.6 million reported cases reported. Currently, it is further spreading to new regions such as European and Eastern Mediterranean.

### 2.4 Yellow fever virus

Yellow fever caused by the yellow fever virus is endemic to tropical regions of Africa and Central and South America, with symptoms ranging from asymptomatic infection to mild illness, fever with jaundice or hemorrhage and death (Garske et al., 2014). There are three main transmission cycles. In the jungle (sylvatic) cycle, YFV is transmitted between non-human primates and mosquitoes. The former serve as the principal reservoirs and amplifying hosts, while the latter act as vectors (Garcia-Oliveira et al., 2023). Furthermore, humans are accidental hosts who can be infected upon entering the jungle. In the urban cycle, the transmission of YFV occurs between humans and mosquitoes. Additionally, in Africa, there exists an intermediate (savannah) cycle in which YFV is transmitted from monkeys to mosquitoes and then to people who live or work in forest-border areas.

Historically, YFV has been responsible for multiple large-scale epidemics over the past few centuries. In 1793, a major yellow fever outbreak in Philadelphia resulted in approximately 4,000 deaths within 3 months, representing nearly 10% of the city’s population at the time (estimated at 55,000) (Rodhain, 2022). Another severe epidemic in the United States in 1878 caused more than 74,000 cases and 16,000 deaths (Rodhain, 2022).

### 2.5 Zika virus

ZIKV was first isolated from a rhesus monkey with fever in 1947 in Uganda (Dick et al., 1952). Before 2007, only sporadic human cases had been reported in countries across Africa and Asia (Hennessey et al., 2016). In 2007, the Zika spread to Yap Island in the Federated States of Micronesia, causing the first documented outbreak; approximately three-quarters of the population was infected, with most cases presenting only mild symptoms, and no reported fatalities (Duffy et al., 2009). Between 2013 and 2014, an outbreak occurred in French Polynesia, a where a subsequent case-control study provided the first evidence linking ZIKV infection to Guillain-Barré syndrome (Cao-Lormeau et al., 2016). In 2015, Brazil reported its first cases of autochthonous Zika transmission, after which the virus spread extensively throughout the Americas (Zanluca et al., 2015; Relich and Loeffelholz, 2017). That same year, in November, the Brazilian Ministry of Health noted an increase in microcephaly among newborns, a rise that coincided geographically and temporally with the ZIKV outbreak (Marrs et al., 2016). Subsequently, accumulating evidence has demonstrated the association between microcephaly and ZIKV infection. From September 2015 to April 2017, Colombia recorded 19,935 suspected ZIKV infections in pregnant women, of whom 157 were associated with neonatal microcephaly (Mattar et al., 2017). In one reported case, a 34-year-old woman exhibited ZIKV symptoms at 19 weeks of gestation; fetal ultrasound revealed multiple abnormalities, including ventriculomegaly and reduced cerebellar volume, and postnatal head CT confirmed microcephaly with intracranial calcifications. In Brazil, the prevalence of microcephaly has been largly attributed to congenital ZIKV infection (De Araújo et al., 2016). Among 87 infants diagnosed with congenital Zika syndrome (CZS) based on abnormal neuroimaging and positive ZIKV-specific IgM in cerebrospinal fluid, 66 of their mothers reported symptoms of ZIKV infection during pregnancy (Meneses et al., 2017). In the United States, 11% of fetuses or infants born to women infected with ZIKV during early pregnancy developed ZIKV-associated birth defects, such as microcephaly accompanied by intracranial calcifications (Honein et al., 2017).

### 2.6 Kyasanur forest disease virus

Kyasanur Forest disease virus (KFDV) was first discovered in 1957 in the Kyasanur Forest of Shimoga District, Karnataka State, India, following an outbreak that cause significant mortality in two local monkey species (Mehla et al., 2009). The primary vector of KFDV is the hard tick Haemaphysalis spinagera (N et al., 2024). Small mammals such as rodents act as amplifying hosts, while larger mammals serve as maintenance hosts. Human infections occur predominantly through tick bites in forested areas with high tick density (N et al., 2024).

The clinical manifestations of Kyasanur Forest disease are typically biphasic. The initial phase is characterized by sudden onset of high fever with chills, accompanied by symptoms such as myalgia, headache, gastrointestinal disturbances, conjunctival congestion, lymphadenopathy, and hepatosplenomegaly (Gupta et al., 2022). Approximately 82%–88% of recovre after this phase, the remaining 12%–18% progress to a second phase characterized by recurrent fever and neurological manifestations (Gupta et al., 2022).

Major human outbreaks were recorded in 1957–1958 (681 cases), 1983–1984 (2,589 cases), 2002–2003 (1,562 cases), and 2016–2017 (809 cases) (Chakraborty et al., 2019). Until 2011, KFDV was restricted to southern India. However, surveillance in 2016 confirmed its geographic expansion into new areas along the Western Ghats, including parts of Karnataka, Tamil Nadu, Kerala, Goa, and Maharashtra.

### 2.7 Tick-borne encephalitis virus

Tick-borne encephalitis virus (TBEV) is endemic across parts of Europe and Asia. Human infection occur through the bite of infected ticks, mainly *Ixodes ricinus* and *Ixodes persulcatu*, leading to tick-borne encephalitis (TBE).

Mostly TBEV infection are asymptomatic. Symptomatic cases may follow either a monophasic or a biphasic clinical course (Kwasnik et al., 2023). He initial phase typically presents with non-specific influenza-like symptoms such as fever, headache, and myalgia. In biphasic cases, a second phase may follow, characterized by neurological signs of encephalitis (Kwasnik et al., 2023). TBEV is classified into three subtypes: European, Siberian, and Far-Eastern. The Far-Eastern subtype generally causes a severe monophasic illness, whereas the European subtype typically exhibits a biphasic progression (Mansfield et al., 2009).

### 2.8 Omsk hemorrhagic fever virus

Omsk Hemorrhagic Fever (OHF) is endemic in certain areas of Siberia. The principal vectors of Omsk Hemorrhagic Fever Virus (OHFV) are ticks–*Dermacentor reticulatus*, *Dermacentor marginatus* and *Ixodes persulcatus*–with muskrats and local voles serving as reservoir hosts (Diani et al., 2025). Human infection occurs mainly via the bite of an infected tick. Incidence peaks between May and June, correlating with the seasonal activity of Dermacentor reticulatus (Diani et al., 2025). Clinical manifestations can include fever, headache, myalgia, and cough, and may progress to hemorrhagic symptoms or meningitis (Diani et al., 2025).

### 2.9 Saint louis encephalitis virus

Human cases of Saint Louis encephalitis virus (SLEV) infection are reported almost exclusively in the United States. SLEV is maintained in an enzootic mosquito-bird transmission cycle, and humans are incidental hosts infected through mosquito bites. The peak transmission period occurs from late summer to early autumn (Ardakani et al., 2024). While most infections are asymptomatic, some individuals develop influenza-like illness; a small proportion progress to neuroinvasive disease, presenting as encephalitis or meningitis (Ardakani et al., 2024).

## 3 IFN system: an overview

### 3.1 IFN classes and functions

Interferons (IFNs), first discovered in 1957, are a group of cytokines that are responsible for antiviral, antitumor and immune regulation (Isaacs and Lindenmann, 1957; Borden et al., 2007). On the basis of their structural features, receptor usage and biological activities, IFNs are grouped into three types: I, II and III (Donnelly and Kotenko, 2010). In humans and mice, type I IFNs include IFN-α, β, ε, κ, ω (humans) and ζ (mice), which bind to and signal through IFNAR, a ubiquitously expressed heterodimeric transmembrane receptor consisting of the IFNAR1 and IFNAR2 subunits (Lazear et al., 2019). In addition, IFN-β can signal through IFNAR1 alone and regulate unique gene expression via non-JAK-STAT-mediated pathway(s) (De Weerd et al., 2013). In contrast, type II IFNs include only IFN-γ, which binds to and signals through the IFNGR complex composed of IFNGR1 and IFNGR2 (Chow and Gale, 2015). In humans, type III IFNs include IFN-λ1 (IL-29), IFN-λ2 (IL-28A), IFN-λ3 (IL-28B) and IFN-λ4, whereas in mice, type III IFNs include only IFN-λ2 and IFN-λ3. All type III IFNs signal through a common heterodimeric receptor known as IFNLR, which consists of IFNLR1 (IL-28Rα) and IL-10R2 (IL-10Rβ) (Lazear et al., 2019). In contrast to ubiquitously expressed IFNAR, IFNLR is expressed preferentially on epithelial cells and neutrophils (Lazear et al., 2019). Specifically, the expression of IL-10R2 is widespread, whereas the expression of IFNLR1, which is associated with the response to IFN III, is confined to epithelial cells, subsets of myeloid cells, and certain neuronal cells (Chow and Gale, 2015; Sommereyns et al., 2008). This expression pattern results in nearly all cells responding to type I IFNs, whereas only a limited subset of cells responds to type III IFNs (Odendall and Kagan, 2015). Consequently, type I IFNs are responsible for establishing a systemic antiviral state, whereas type III IFNs control infection mainly at barriers, including the respiratory and gastrointestinal tracts, the blood-brain barrier (BBB) and placental trophoblasts (Wells and Coyne, 2018). In this review, we concentrate on the antiviral functions of type I and III IFNs at the BBB and placental barrier.

### 3.2 Activation of IFN expression

**FIGURE 2:**
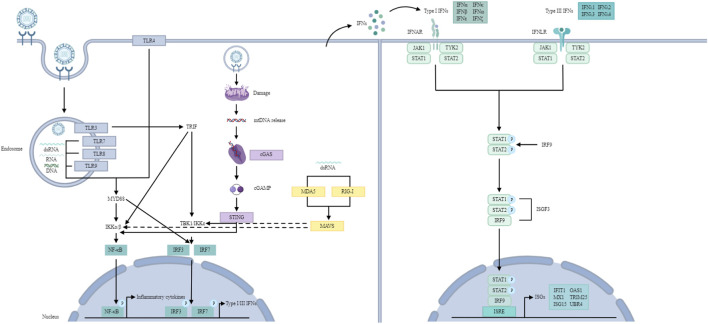
IFN signalling pathway. TLR signalling pathway: TLRs recognize dsRNA, ssRNA and unmethylated CpG DNA. TLR3 and TLR4 utilize the TRIF-dependent pathway to activate IRF3 and NF-κB, inducing type I IFNs and inflammatory cytokines. Other TLRs (with the exception of TLR3) utilize the MYD88-dependent pathway to activate NF-κB and inflammatory cytokines. TLR7-MYD88 signalling induces IFNs through the phosphorylation and translocation of IRF7. RLR signalling pathway: RLRs recognize dsRNA. RIG-I and MDA5 then activate MAVS, which recruits and activates TBK1 and IKK to phosphorylate IRF3/7 and NF-κB, resulting in type I and III IFN production. cGAS/STING pathway: cGAS recognizes mtDNA. Later, cGAS leads to cGAMP synthesis, which activates STING, resulting in the activation of TBK1 and IKK, ultimately producing type I IFNs. JAK/STAT pathway: Type I and III IFNs bind to their receptors, activating JAK1 and TYK2, leading to the formation of ISGF3, which translocates to the nucleus and binds to ISREs, inducing ISGs.

Pattern recognition receptors (PRRs), which recognize pathogen-associated molecular patterns (PAMPs) and damage-associated molecular patterns (DAMPs), along with their associated signalling pathways, constitute a large part of the innate immune system (Carty et al., 2021; Wicherska-Pawłowska et al., 2021). PRRs include Toll-like receptors (TLRs), retinoic acid-inducible gene-I (RIG-I)-like receptors (RLRs), NOD-like receptors (NLRs) and cytosolic DNA sensors such as cyclic GMP-AMP synthase (cGAS) (Cao, 2016) (Figure 2). TLRs recognize double-stranded RNA (dsRNA), single-stranded RNA (ssRNA) or unmethylated CpG DNA (Onomoto et al., 2021). While RLRs sense viral RNA in the cytoplasm, cGAS is a DNA sensor (Kim and Song, 2022; Sun et al., 2013). Generally, RNA viruses are detected in the endosomal compartment by TLRs or in the cytoplasm by RLRs (Park and Iwasaki, 2020). Once PAMPs are sensed, PRRs trigger a signalling cascade that results in the production of IFNs, inflammatory cytokines and chemokines to establish an immune response (Gürtler and Bowie, 2013).

#### 3.2.1 TLRs

TLRs are extensively expressed on the cell surface or in endosomal membranes of effector cells and serve as intermediates that interact with viral replication products and transmit signals to a series of adapters and kinases, leading to the transcriptional activation of cytokines and type I interferon genes (Hu et al., 2023; Zeng et al., 2023a). To date, 10 types of TLRs have been identified in humans, of which TLR3 and TLR4 utilize the TRIF-dependent pathway to activate the transcription factors interferon regulatory factor 3 (IRF3) and nuclear factor-κB (NF-κB) and then induce type I IFNs and inflammatory cytokines (Kawai and Akira, 2010). In contrast, all TLRs except TLR3 utilize the MYD88-dependent pathway to activate NF-κB and mitogen-activated protein kinases (MAPKs) to induce inflammatory cytokines (Cao, 2016). In addition, TLR7-MYD88 signalling induces IFNs through the phosphorylation and translocation of IRF7 (Van Der Sluis et al., 2022). Notably, adaptors (MYD88 and TRIF) activate IRFs and NF-κB by recruiting TANK-binding kinase 1 (TBK1) and inhibitory κB kinase (IKK) (Gewaid and Bowie, 2024). In many cases, NLRs appear to have an inhibitory effect on TLR signalling (Cao, 2016).

#### 3.2.2 RLRs

RLRs include retinoic acid-inducible gene I (RIG-I), melanoma differentiation-associated antigen 5 (MDA5), and laboratory of genetics and physiology 2 (LGP2) (Yoneyama and Fujita, 2007). Both RIG-I and MDA5 detect viral RNA and produce IFNs upon infection (Lu et al., 2022). All RLRs contain a DExD/H-box RNA helicase domain and a C-terminal domain (CTD), whereas RIG-I and MDA5, rather than LGP2, have two N-terminal tandemly linked caspase activation and recruitment domains (CARDs), which interact with mitochondrial antiviral signalling protein (MAVS/IPS-1) (Yoneyama et al., 2015). Activated MAVS recruits and activates TBK1 and IKK, which then phosphorylate IRF3/7 and NF-κB to generate type I and III IFNs (Labib and Chigbu, 2022).

#### 3.2.3 cGAS-STING

In fact, the cGAS-STING pathway also restricts ortho-flavivirus infection. DENV infection triggers the release of mitochondrial DNA (mtDNA) into the cytoplasm, which is then detected by cGAS and triggers the cGAS/STING pathway to produce IFN-I (Aguirre et al., 2017; Aguirre and Fernandez-Sesma, 2017). Specifically, upon detection, cGAS dimerizes and catalyzes the synthesis of 2′,3′-cyclic GMP-AMP (cGAMP), which is recognized by STING as the second messenger, leading to the activation of TBK1 and IKK (Zoladek and Nisole, 2023).

In summary, upon activation by infection, these PRRs initiate two primary signalling pathways: the NF-κB pathway is responsible for driving the production of inflammatory cytokines, whereas the IRF3/IRF7 pathway promotes the expression of IFNs (Zoladek and Nisole, 2023) (Figure 2). Notably, the production of IFNs requires the phosphorylation of IRF3 and IRF7, a process that relies on the activation of TBK1 and IKK.

### 3.3 IFN signalling

All IFNs signal through the JAK/STAT pathway (Chow and Gale, 2015) (Figure 2). Typically, the binding of type I and III IFNs to their respective receptors activates JAK1 and TYK2, resulting in STAT1-2 heterodimerization and interferon-stimulated gene factor 3 (ISGF3) formation (Chow and Gale, 2015). Activated ISGF3 translocates to the nucleus and binds to IFN-stimulated response elements (ISREs) to induce the transcription of ISGs (Schoggins, 2019). ISG effectors target different steps in the viral replication cycle, including viral entry, viral genome nuclear import, viral gene or protein synthesis, viral genome replication, and virion assembly/egression, to perform antiviral functions (Schoggins, 2019).

## 4 Orthoflavivirus evasion of the IFN response

**FIGURE 3:**
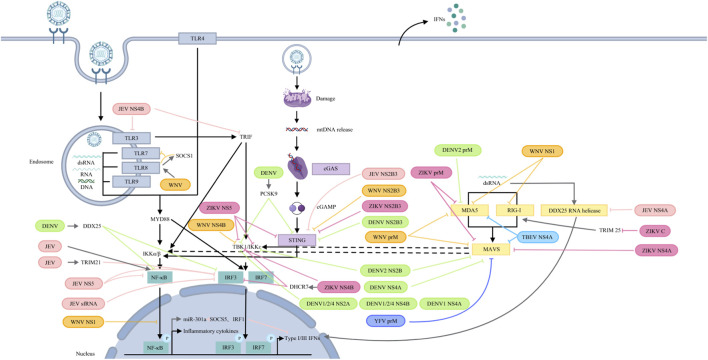
Orthoflaviviruses target PRRs and their signalling pathways. JEV: NS5 blocks IRF3 and NF-κB nuclear translocation; NS4B targets TLR3 and TRIF; and NS4A inhibits DDX42 RNA helicase activity. JEV infection induces TRIM21, attenuating IRF3 phosphorylation. JEV sfRNA inhibits IRF3 phosphorylation and nuclear translocation. JEV activates NF-κB to induce miR-301a, which reduces IRF1 and SOCS5 to antagonize IFN-β production. NS2B3 cleaves STING. WNV: prM interacts with MDA5 and MAVS; NS1 interacts with and degrades RIG-I and MDA5 and suppresses NF-κB activation; NS4 blocks TBK1 phosphorylation and activation. TLR8 signalling upregulates SOCS1, which couples with TLR8 to inhibit TLR7 and ISG56. NS2B3 cleaves STING. DENV: DENV2 prM interacts with MDA5, while NS2B binds to MAVS and IKK. DENV 1/2/4 NS2A and NS4B block TBK1 activation. DENV1 NS4A inhibits TBK1-directed IFN production. DENV NS4A binds to MAVS. DENV NS2B3 cleaves STING. DENV infection increases PCSK9 to inhibit STING and TBK1 phosphorylation and upregulates DDX25 to block IRF3 and NF-κB activation. YFV: prM binds to MAVS. ZIKV: prM binds to MDA5 and MAVS. C interacts with TRIM25 to inhibit RIG-I ubiquitination. NS4A interacts with MAVS. NS4B binds directly to TBK1 and upregulates DHCR7 to inhibit TBK1 and IRF3 phosphorylation. NS5 interacts with TBK1, IKKɛ and STING. NS2B3 cleaves STING. TBEV: prM hinders the interaction between MDA5 and MAVS.

**FIGURE 4:**
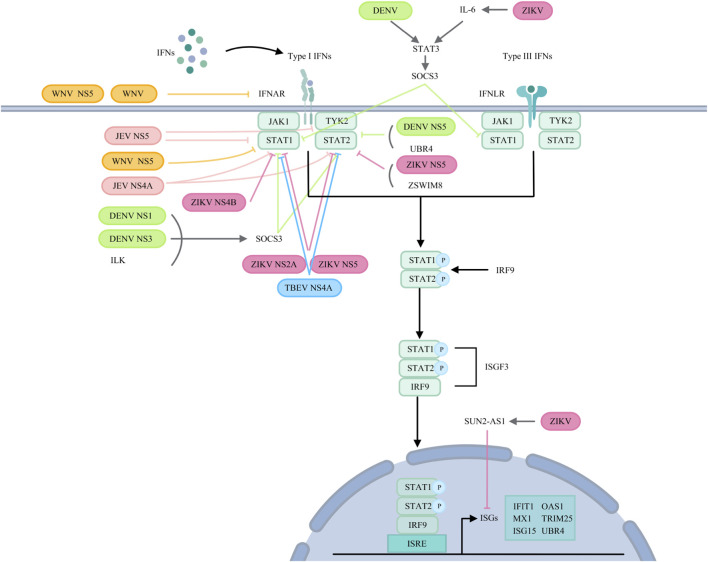
Orthoflaviviruses target IFN signalling pathways. JEV: JEV NS5 suppresses TYK2 and STAT1 activation and inhibits STAT1 phosphorylation. NS4A affects STAT1 and STAT2 phosphorylation. WNV: WNV reduces IFNAR1 levels via noncanonical protein degradation or NS5. NS5 prevents pY-STAT1 accumulation. DENV: ILK interacts with NS1 and NS3 to upregulate SOCS3, inhibiting STAT1 and STAT2 phosphorylation. DENV induces STAT3 to upregulate SOCS3, thereby negatively regulating STAT1 activation. NS5 binds to UBR4 to degrade STAT2. ZIKV: NS2A degrades STAT1 and STAT2. NS4B inhibits STAT1 phosphorylation. NS5 reduces STAT2 levels and inhibits STAT1 phosphorylation. ZIKV infection induces SUN2-AS1 to downregulate ISG expression. ZIKV infection also induces IL-6, which helps phosphorylate STAT3 and transcribe SOCS3, inhibiting STAT1 phosphorylation. ZIKV NS5 utilizes ZSWIM8 to degrade STAT2. TBEV: NS4A blocks STAT1/STAT2 phosphorylation and dimerization.

Despite the host’s ability to induce an antiviral state via interferon, orthoflaviviruses employ multiple sophisticated strategies to evade the interferon response and attenuate host immunity. These mechanisms often involve interference with key components of immune signaling pathways (Figures 3, 4).

### 4.1 Orthoflaviviruses target PRRs and their signalling pathways

#### 4.1.1 JEV

JEV utilizes several non-structural proteins to subvert PRR signaling. The NS5 protein competitively inhibits the interaction of IRF3 and NF-κB with nuclear transport proteins, thereby blocking their dsRNA-induced nuclear translocation and suppressing type I IFN induction (Ye et al., 2017). JEV NS4B targets TLR3 and TRIF to inhibit IFN-β production (Zeng et al., 2023b). While NS4A interacts with the RNA helicase, DDX42—a potential dsRNA sensor—and inhibits its activity, thereby reducing IFN-β induction (Lin et al., 2008). In human microglial (CHME3) cells, JEV infection upregulateds TRIM21 which attenuates IRF3 phosphorylation and IFN-β production (Manocha et al., 2014). Additionly, JEV-derived short fragment ncRNA (sfRNA) impairs IRF3 phosphorylation and nuclear translocation (Chang et al., 2013). Through NF-κB, JEV also induces miR-301a which downregulates IRF1 and suppressor of cytokine signalling 5 (SOCS5), further antagonzing IFN-β induction (Hazra et al., 2017).

#### 4.1.2 WNV

WNV employs its prM protein to interact with MDA5 and MAVS, thereby inhibiting RLR-mediated IFN-I induction (Sui et al., 2023). The NS1 protein binds to and promotes the degradation of RIG-I and MDA5, and specifically reduces K63-linked polyubiquitination of RIG-I, suppressing IFN-β production (Zhang et al., 2017). WNV NS1 also impairs TLR3-mediated activation of the IFN-β promoter and NF-κB-responsive promoters (Wilson et al., 2008). Furthermore, NS4B blocks TBK1 phosphorylation and activation (Dalrymple et al., 2015). Upregulation of SOCS1 via TLR8 signaling during WNV infection inhibits the expression of TLR7 and ISG56 (Paul et al., 2016).

#### 4.1.3 DENV

DENV serotype 2 (DENV2) prM interacts with MDA5 to inhibit IFN-I production (Sui et al., 2023). Moreover, DENV2 NS2B binds to MAVS and IKK to interrupt RLR signalling (Nie et al., 2023). For DENV1, 2, and 4, NS2A and NS4B block TBK1 activation, while DENV1 NS4A also inhibits TBK1-directed IFN induction (Dalrymple et al., 2015). DENV NS4A also binds to MAVS, preventing RIG-I-MAVS complex formation (He et al., 2016). The NS2B3 protease cleaves human STING, antagonizing IFN-I protuction (Aguirre et al., 2012). Under hypoxic conditions, DENV upregulates PCSK9, which inhibits phosphorylation of STING and TBK1 (Gan et al., 2020). DENV infection also upregulates DDX25, which impairs IRF3 and NF-κB activation and IFN-I production (Feng et al., 2017). YFV prM can bind to MAVS to inhibit IFN-I production (Sui et al., 2023).

#### 4.1.4 ZIKV

ZIKV prM binds to both MDA5 and MAVS and prevents the formation of the MDA5-MAVS complex to antagonize IFN-I production (Sui et al., 2023). The capsid protein binds TRIM25 and inhibits its ubiquitination of RIG-I (Airo et al., 2022). ZIKV NS4A interacts with MAVS, blocking ccess for MDA5/RIG-I and disrupting RLR signaling; both the N-terminal CARD-like and C-terminal transmembrane domains of MAVS are involved (Ma et al., 2018; Hu et al., 2019). ZIKV NS4A also mimics phosphorylated IRF3 and broadly inhibits MDA5/RIG-I signaling components (Ngueyen et al., 2019). NS2A similarly downregulates multiple factors in this pathway (Ngueyen et al., 2019). ZIKV NS4B directly binds to TBK1, impairing IFN-β production (Sarratea et al., 2023), and indirectly suppresses it by upregulating DHCR7, which inhibits TBK1 and IRF3 phosphorylation (Chen et al., 2023). ZIKV NS5 interacts with TBK1 and IKKε, inhibiting IRF3 phosphorylation and IFN-I promoter acticvation (Lin et al., 2019; Lundberg et al., 2019). A conserved active site (D146) in ZIKV NS5 is critical for suppressing both RIG-I and cGAS-STING signaling (Li et al., 2020; Li et al., 2024). While NS5 promotes STING cleavage via K48-linked polyubiquitination, the NS2B3 protease directly cleaves STING—a mechanism shared with DENV, JEV, and WNV (Aguirre et al., 2012; Li et al., 2024; Ding et al., 2018).

#### 4.1.5 Other flaviviruses

The structural protein prM of TBEV functions as a key viral antagonist of the innate immune response. It specifically impedes the critical interaction between the cytosolic RNA sensor MDA5 and its downstream adaptor protein MAVS. By disrupting the formation of the MDA5-MAVS complex, TBEV prM effectively blocks the subsequent signaling cascade that leads to the activation of transcription factors IRF3 and IRF7, thereby suppressing the production of type I IFNs (Sui et al., 2023).

### 4.2 Orthoflaviviruses target IFN signalling pathways

#### 4.2.1 JEV

JEV NS5 suppresses the activation of TYK2 and STAT1 to block the IFN-α signalling (Lin et al., 2006), and inhibits the IFNβ‐induced phosphorylation of STAT1 at Tyr701 (Yang et al., 2013). JEV NS4A blocks the JAK-STAT signalling pathway by affecting the phosphorylation of STAT1 and STAT2 (Lin et al., 2008). JEV also modulates SOCS1 and SOCS3 expression to affect the JAK-STAT signalling cascade (Kundu et al., 2013).

#### 4.2.2 WNV

WNV reduces IFNAR1 protein levels via a noncanonical protein degradation pathway, attenuating IFN response (Evans et al., 2011). The NS5 protein binds prolidase (PEPD), which is essential for IFNAR1 maturation, thereby downregulating IFNAR1 and impairing IFN-I-dependent antiviral gene induction (Lubick et al., 2015). WNV NS5 from virulent NY99 strain prevents STAT1 phosphorylation and suppresses ISGs expression (Laurent-Rolle et al., 2010).

#### 4.2.3 DENV

In DENV-2 infection, upregulation of USP18 blunts the antiviral effect of IFN-α, whereas USP18 silencing enhances JAK-STAT signaling (Ye et al., 2021). Alternatively, USP18 competes with DENV and ZIKV NS5 for STAT2 binding, and ISG15 stabilizes USP18 to protect STAT2 from NS5-mediated degradation (Espada et al., 2024). DENV also induces STAT3 phosphorylation, upregulating SOCS3, which negatively regulates STAT1 and antagonizes IFN-I and IFN-III responses (Srivastava et al., 2021). Furthermore, integrin-linked kinase (ILK) interacts with DENV NS1 and NS3 to promote SOCS3 expression via the Akt-Erk-NF-κB pathway, inhibiting STAT1/2 phosphorylation and ISG expression (Kao et al., 2023). DENV NS5 binds STAT2 and induces its degradation in a UBR4-dependent manner (Ashour et al., 2009), whereas ZIKV-induced STAT2 degradation relies on ZSWIM8 (Morrison et al., 2013; Grant et al., 2016; Ren et al., 2024).

#### 4.2.4 ZIKV

ZIKV INMI1 strain exploits NS2A protein to degrade both STAT1 and STAT2, inhibiting IFN-I and IFN-II signalling (Fanunza et al., 2021a). The same strainemploys NS4B to inhibit STAT1 phosphorylation and block nuclear translocation of phosphorylated STAT2 (Fanunza et al., 2021b). The epidemic Brazilian ZIKV NS5 protein reduces STAT2 levels and inhibits STAT1 phosphorylation (Hertzog et al., 2018). ZIKV infection also induces the lncRNA SUN2-AS1, which downregulates ISGs and facilitates viral replication (Yang et al., 2024). Furthermore, AXL attenuates IFN signaling by regulating SOCS1 in a STAT1/STAT2-dependent manner, rather than acting primarily as an entry receptor (Chen et al., 2018). ZIKV-activated TLR3 triggers IL-6 production, leading to STAT3 phosphorylation and SOCS3 transcription, which in turn inhibits STAT1 phosphorylation and RLR-induced IFN responses (Plociennikowska et al., 2021).

#### 4.2.5 Other flaviviruses

Beyond the major flaviviruses, other members of the orthoflavivirus genus also employ specific viral proteins to disrupt type I IFN signaling. The NS5 of KFDV, particularly within its RNA-dependent RNA polymerase (RdRp) domain, functions as the principal viral effector that antagonizes the JAK-STAT pathway. It potently inhibits the phosphorylation and nuclear translocation of key signal transducers, thereby blocking the downstream transcriptional activation of ISGs and compromising the host’s antiviral state (Cook et al., 2012). Similarly, TBEV utilizes its NS4A protein to effectively hinder the JAK-STAT cascade. TBEV NS4A directly interferes with the phosphorylation and subsequent dimerization of STAT1 and STAT2, critical steps for the formation of the transcriptional complex ISGF3. This blockade prevents the expression of a broad spectrum of ISGs, facilitating viral immune evasion and persistence within the host (Yang et al., 2020). These mechanisms underscore the convergent evolution among diverse flaviviruses to target the core of the IFN-mediated antiviral defense.

## 5 Interaction of orthoflaviviruses and IFNs at the barrier surface

The interaction between flaviviruses and type I/III interferons at the blood-brain and placental barriers represents a scientifically significant area of investigation, with importance manifested in two key aspects. First, these barriers serve as critical gateways determining viral neuroinvasiveness and vertical transmission potential. Understanding how flaviviruses overcome or exploit local interferon responses is essential for elucidating the pathogenic mechanisms underlying flavivirus-induced neurological disorders and congenital syndromes. Second, type III interferons function as specialized “sentinels” at barrier surfaces, exhibiting both collaborative and distinct mechanisms compared to the systemically active type I interferons. Delineating this functional specialization and cooperation will not only advance our knowledge of host-virus interactions within specific microenvironments but may also provide a theoretical foundation for developing targeted immunomodulatory strategies.

### 5.1 Placental barrier

**FIGURE 5:**
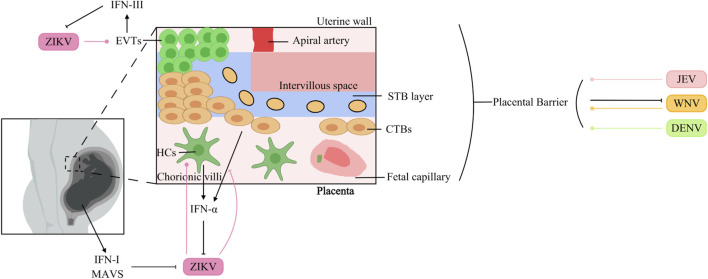
Interaction of Orthoflaviviruses and IFNs at the placental barrier surface. JEV: JEV infects human endometrial epithelial and trophoblast cells. WNV: WNV infects the placenta, but differentiated STBs protect against WNV infection. DENV: DENV can cross the placenta to infect the fetal circulation. ZIKV: Fetal MAVS and IFN-I can restrict placental infection. ZIKV infects HCs, which in turn increases IFN-α production. ZIKV surpasses the barrier composed of CTBs and inhibits IFN-α production in HTR8 cells. EVTs induce IFN-III production during ZIKV infection, which suppresses ZIKV replication.

The placental barrier is a specialized biological interface separating maternal and fetal circulations. It is consists of a multilayered membrane structure comprising diverse cellular components derived from both maternal and fetal tissues (Arumugasaamy et al., 2020; Levkovitz et al., 2013). Maternal contributions include decidual stromal cells, whereas fetal-derived populations include trophoblast lineages (villous cytotrophoblasts [VCTs], syncytiotrophoblasts [STBs], extravillous trophoblasts [EVTs], and trophoblast giant cells [TGCs]) alongside nontrophoblastic cells such as Hofbauer macrophages and fetal endothelial cells (Arumugasaamy et al., 2020). This dynamic interface acts as a bidirectional regulatory system, coordinating nutrient transport, hormone synthesis, and growth factor secretion essential for fetal development, while also facilitating waste elimination and limiting fetal exposure to xenobiotics (Arumugasaamy et al., 2020). Beyond its metabolic and protective functions, the placenta serves as a critical immunological sentinel, deploying multiple defence mechanisms against microbial invasion. However, certain pathogens, collectively referred to as TORCH agents (including Toxoplasma gondii, rubella virus, cytomegalovirus, herpes simplex virus, syphilis, Zika virus, Plasmodium spp., and HIV), have evolved strategies to bypass these defences, often resulting in severe congenital infections that can lead to fetal demise or lifelong morbidity (Lynn et al., 2023). The mechanisms by which flaviviruses traverse the placental barrier have been extensively studied (Figure 5).

#### 5.1.1 JEV

JEV causes persistent infection in the human endometrial epithelium and trophoblast, probably facilitating transplacental JEV transmission (Chapagain et al., 2022). Moreover, an *in vitro* transwell model demonstrated that extracellular vesicles (EVs) purified from JEV-infected cells promoted the crossing of the placental barrier by JEV (Xiong et al., 2025).

#### 5.1.2 WNV

Maternal infection with WNV during pregnancy can result in vertical transmission and subsequent fetal infection. This phenomenon is intricately linked to the developmental status of the blood-fetal barrier at the time of infection. In mouse fetuses, dams infected with WNV at 7.5 days post-coitus (dpc) presented a significantly higher rate of maternal-to-fetal viral transmission than did those infected at 11.5 dpc, where fetal infection occurred less frequently. The placental barrier is established in mice at approximately 10.5 dpc, demonstrating that the placenta serves as a protective barrier against WNV infection (Julan et al., 2006). The precise molecular mechanisms underlying placental resistance to WNV infection remain incompletely understood.

#### 5.1.3 DENV

Studies employing distinct mouse models and viral strains have yielded opposite conclusions. Watanabe et al. reported that DENV can cross the placental barrier in AG129 mice (Watanabe et al., 2022). However, Zhang et al. demonstrated that in IFNAR1^−/−^ mice, DENV-2 cannot infect fetuses through transplacental transmission, and the fetal intrauterine growth restriction (IUGR) caused by maternal DENV-2 infection can be attributed to neutrophil infiltration into the placenta and the destructive effects of this infiltration on the placental vasculature (Zhang Y. et al., 2023).

#### 5.1.4 ZIKV

ZIKV is now recognized as a TORCH pathogen, though the exact mechanisms of placental crossing are not fully understood. In immunocompetent mice, midgestation (E9.5) intravenous injection of ZIKV led to infection of the decidua and placenta, followed by fetal growth restriction (Alippe et al., 2024). Fetal restriction of placental infection was mediated by MAVS and type I IFN signaling, rather than by TLR7, TLR9, MyD88, STING, or type III IFN pathways (Alippe et al., 2024). *In vitro*, ZIKV infects and replicates in Hofbauer cells (HCs) and cytotrophoblasts (CTBs), which subsequently mount an antiviral response—including upregulation of IFN-α in HCs (Quicke et al., 2016). Viettri et al. further demonstrated that ZIKV, unlike DENV and YFV, can traverse a CTB-based barrier; once it enters the placental stroma, it targets HCs, enabling spread to the fetal circulation (Viettri et al., 2023). The same group reported that ZIKV infection inhibits IFN-α production in HTR8 cells (derived from CTBs) (Viettri et al., 2023). Conversely, first-trimester trophoblast cells upregulate IFN-β and ISG expression upon ZIKV infection (Ding et al., 2021). Additionally, trophoblast stem cell-derived trophoblasts infected with ZIKV release IFNs that protect embryonic stem cells via paracrine signaling in the blastocyst (Fendereski et al., 2022). Type III IFNs also contribute to placental anti-ZIKV defence: STBs from the human term placenta release IFN-λ1, protecting both trophoblast and nontrophoblast cells through autocrine and paracrine mechanisms (Bayer et al., 2016). Human maternal decidual tissues upregulate both type I and type III IFNs in response to ZIKV infection (Weisblum et al., 2017), and recombinant IFN-λ inhibits ZIKV infection in human midgestation maternal-fetal explants (Jagger et al., 2017). In mice, mid-pregnancy treatment with IFN-λ improved fetal growth restriction and suppressed ZIKV replication (Chen et al., 2017). More recently, Azamor et al. showed that term decidual EVTs also produce IFN-λ upon ZIKV infection (Azamor et al., 2024). Notably, IFNs can also exert pathogenic effects during pregnancy. Fetal IFNAR signaling, while controlling ZIKV replication in the placenta, contributes to adverse outcomes such as IUGR and fetal resorption (Yockey et al., 2018). In mouse models of congenital ZIKV infection, maternal type III IFN administration at E7 caused detrimental effects, whereas the same treatment at E9 protected against transplacental viral transmission (Casazza et al., 2022).

### 5.2 Blood-brain barrier

**FIGURE 6:**
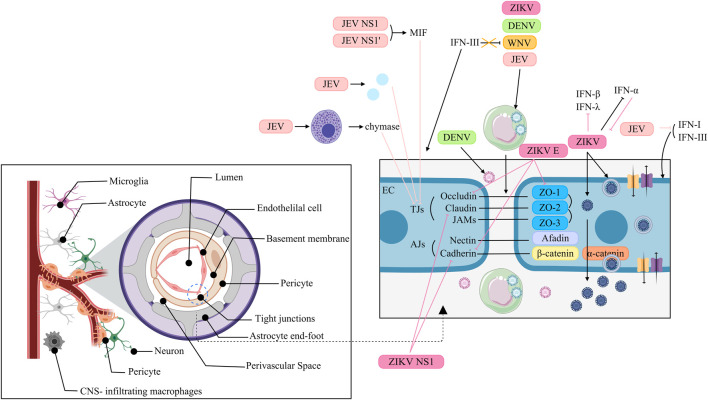
Interaction of Orthoflaviviruses and IFNs at the blood-brain barrier. JEV: NS1 and NS1 induce MIF to degrade TJs. JEV activates MCs to release chymase, promoting TJ cleavage. JEV upregulates inflammatory cytokines/chemokines to suppress TJ expression. JEV uses “Trojan horses” to traverse the BBB. IFN-I and IFN-III help stabilize the BMEC barrier, but JEV represses these IFNs. WNV: WNV exploits “Trojan horses” to traverse the BBB. IFN-III fails to suppress WNV infection but strengthens BBB integrity. DENV: DENV reaches the CNS via paracellular transport and uses “Trojan horses”. ZIKV: ZIKV crosses the BBB via transcytosis. HBMECs infected with ZIKV serve as viral reservoirs, allowing ZIKV to be released into the neuronal compartments. ZIKV regulates IFN-β and -λ expression. IFN-α reduces ZIKV infection, but ZIKV inhibits this antiviral function. ZIKV uses “Trojan horses” to traverse the BBB. ZIKV E downregulates ZO-1, VE-Cadherin and Occludin and alters their localization. ZIKV NS1 inhibits VE-cadherin and claudin-5 expression.

The BBB is a physical barrier in the CNS that separates the CNS parenchyma from the peripheral blood, thereby maintaining the normal physiology of the brain. However, the BBB is not a single physical structure but rather represents the comprehensive effect of various physiological characteristics possessed by endothelial cells (ECs), which collectively restrict vascular permeability (Profaci et al., 2020). Specifically, the restricted paracellular permeability of the capillary EC layer is achieved through adherens junctions (AJs) and tight junctions (TJs) (Erdő et al., 2017). AJs maintain adhesion between ECs and are composed of two transmembrane components: cadherins and nectins (Campbell et al., 2017). In adjacent cells, cadherins bind to each other, and the cytoplasmic tail of cadherin recruits β-catenin, which binds to α-catenin (Campbell et al., 2017). Similarly, the cytoplasmic tail of nectin recruits afadin, while the extracellular regions dimerize with those on neighboring cells (Campbell et al., 2017). In ECs, the most apical intercellular junctions are the TJs (Erdő et al., 2017). TJs have two functions, which are supposed to be mutually exclusive (Hartsock and Nelson, 2008). One is the barrier or gate function, which controls the paracellular passage of ions, water and macromolecules (Campbell et al., 2017; Hartsock and Nelson, 2008). Another is the fence function, which restricts lipid distribution within the membrane to establish and maintain cell polarity (Campbell et al., 2017). TJs are composed of claudins, occludin, ZO proteins and junctional adhesion molecules (JAMs) (Campbell et al., 2017). In addition to ECs, the cellular components of the BBB include pericytes, astrocytes, microglia, and neurons. The interaction between ECs and these cells is typically known as the neurovascular unit (NVU) (Profaci et al., 2020). In addition to its cellular components, the BBB consists of the extracellular matrix and basal lamina, which serve as part of the protective system (Erdő et al., 2017). Despite the protective role of the intact BBB in safeguarding the CNS against viral infections, certain ortho-flaviviruses can traverse the BBB via diverse mechanisms, thereby infecting neurons and inducing a spectrum of manifestations (Figure 6).

#### 5.2.1 JEV

JEV can disrupt the BBB through a variety of mechanisms. JEV NS1 and NS1’ proteins induce the expression of macrophage migration inhibitory factor (MIF), which then induces the degradation of TJs through the autophagy-lysosomal pathway, resulting in disruption of the BBB (Zhang et al., 2024). Additionally, JEV might increase the permeability of the BBB via hypermethylation of the TJ gene *Afdn* promoter, which in turn downregulates the expression of AFDN (Xiang et al., 2024). Moreover, JEV activates mast cells (MCs) to release granule-associated proteases, especially chymase, which promotes BBB breakdown and TJ protein cleavage (Hsieh et al., 2019). Furthermore, the modulation of the permeability of the BBB does not directly result from JEV itself but from the inflammatory cytokines/chemokines upregulated by JEV infection in the CNS, which in turn suppresses the expression of TJs (Li et al., 2015). Hence, JEV can pass through the BBB without disrupting it. In addition, JEV can traverse the BBB by exploiting infected monocytes as “Trojan horses” (Zou et al., 2021). Interferons contribute to BBB stabilization during JEV infection; both type I and III IFNs have been shown to reinforce the brain microvascular endothelial cell (BMEC) barrier (Zhang Y. G. et al., 2023). However, JEV counteracts this defense by activating the EGFR–ERK signaling cascade, which suppresses host IFN signaling and promotes viral replication in human BMECs (Zhang et al., 2022).

#### 5.2.2 WNV

The mechanisms that WNV utilizes to infect the CNS might involve a temporal process. Initially, cell-free WNV can migrate without affecting BBB permeability (Verma et al., 2009). Later, WNV replicates in the CNS, which breaks up the BBB, thereby contributing to viral neuroinvasion through the “Trojan horse” route (Roe et al., 2012). IFN signaling plays a restorative role: although IFN-III does not directly suppress WNV replication, it enhances BBB integrity, thereby limiting viral neuroinvasiveness (Lazear et al., 2015).

#### 5.2.3 DENV

DENV adopts stage-dependent mechanisms to access the CNS. In early infection, viral uncoating, transcription, and translation contribute to BBB disruption, allowing paracellular viral entry. In later stages, DENV primarily relies on the “Trojan horse” mechanism to infiltrate the nervous system (Velandia-Romero et al., 2016).

#### 5.2.4 ZIKV

ZIKV penetrates the BBB to infect neural cells, likely via a transcellular mechanism, without compromising barrier integrity (Alimonti et al., 2018). Persistently infected hBMECs may serve as viral reservoirs, releasing ZIKV basolaterally into neuronal compartments (Chiu et al., 2020). During infection, ZIKV strains such as PRVABC59 induce transcription of IFN-β and IFN-λ, but these cytokines are undetectable in supernatants, suggesting post-transcriptional regulation of expression or secretion (Mladinich et al., 2017). This discrepancy is attributed to ZIKV-mediated suppression of RPS6 phosphorylation, which inhibits IFN-β translation (Wang et al., 2023). Although exogenous IFN-α reduces ZIKV infection, the virus rapidly develops resistance (Mladinich et al., 2017). ZIKV may also cross the BBB via infected extracellular vesicles (iEVs) acting as “Trojan horses” (Fi et al., 2021). Furthermore, ZIKV directly impairs BBB integrity: the E protein downregulates and mislocalizes ZO-1, VE-cadherin, and occludin in hBMECs (Kaur et al., 2023). While NS1 suppresses VE-cadherin and claudin-5 expression through microRNA-mediated pathways (Bhardwaj and Singh, 2021; Bhardwaj and Singh, 2023).

## 6 Research limitations

As previously noted, numerous studies have explored the interactions between flaviviruses and IFNs at the PB and BBB. However, the majority of these findings are based exclusively on *in vitro* research. For example, JEV has been shown to cross the placenta, whereas ZIKV can infect HCs as well as CTBs and trigger the production of IFNs (Chapagain et al., 2022; Xiong et al., 2025; Quicke et al., 2016). This reliance on *in vitro* models without corresponding validation *in vivo* is especially evident in BBB research. A substantial body of research suggests that flaviviruses may gain access to the central nervous system via transcellular transport, paracellular diffusion, the “Trojan horse” route, or compromised barrier integrity. However, the extent to which these mechanisms operate under physiological conditions remains unclear and requires confirmation in living systems.

Indeed, the primary target cells for flavivirus infection *in vivo* may differ significantly from those used in vitro models. Notably, *in vivo* studies have demonstrated that WNV predominantly infects neuronal cells in the mouse brain rather than the BMECs commonly employed *in vitro* (Suen et al., 2014). Therefore, although *in vitro* experiments provide valuable insights into potential viral pathways, they often fail to recapitulate the complexity of physiological and pathological processes in intact organisms. This discrepancy raises doubts about the reliability of the current findings and highlights the need for more comprehensive *in vivo* investigations.

## 7 Treatment

Although the lack of *in vivo* evidence underscores the limitations noted above, animal models have preliminarily revealed a central protective role of interferon signaling, pointing to a viable therapeutic direction. For instance, IFN-α/β receptor-deficient (A129) mice show detectable DENV in the brain as early as 3 days post-infection, whereas immunocompetent 129/Sv/Ev mice do not (Shresta et al., 2004). Similarly, A129 mice exhibit elevated YFV load in the brain 4 days after infection, unlike wild-type controls (Meier et al., 2009). ZIKV is detected in the brains of A129 mice by day three, whereas 129/Sv/Ev mice show no infection until day seven (Dowall et al., 2016). Moreover, ABR^−/−^ mice demonstrate increased mortality following SLEV infection (Rocha et al., 2021). Toghter, these findings highlight the essential role of intact IFN signaling in conferring CNS resistance to flaviviruses.

Sporadic human studies have further supported the therapeutic potential of interferons against flaviviral infections. As early as 2004, experiments indicated that early initiation of IFN-α2b therapy could reduce the severity and duration of complications in SLEV-induced meningoencephalitis (Rahal et al., 2004). In solid organ transplant (SOT) recipients infected with SLEV, delayed administration of IFN combined with intravenous immunoglobulin (IVIG) appeared to alleviate neurological symptoms (Hartmann et al., 2017). Similarly, in SOT recipients infected with WNV, IVIG alone or combined with IFN improved clinical symptoms in 70% of patients (16 of 23), including four who achieved complete recovery (Kasule et al., 2023).

Although interferon-based therapies remain under investigation, agents from other drug classes are steadily advancing into clinical trials. For example, AT-752, a guanine nucleotide prodrug inhibitor against DENV, has already entered Phase II trials (Horga et al., 2025). In the Phase II/III trial among DENV adult patients, oral Ivermectin accelerated the clearance of NS1 antigen (Suputtamongkol et al., 2021). NmAb MBL-YFV-01 can protect rhesus macaques from death after YFV infection (Rust et al., 2025). In the Phase I trial, TY014 reduced the viremia and associated symptoms induced by YF17D (Low et al., 2020).

Collectively, interferon-based regimens exhibit multilayered therapeutic potential against flaviviral infections. First, they exert direct antiviral activity: flaviviruses sustain genomic replication and precipitate clinical manifestations by downregulating host IFN signalling and attendant effector molecules, exogenous IFN can counteract this suppression, thereby reducing viremia and ameliorating symptoms. Second, IFNs mitigate infection-driven pathological injury. Flaviviruses compromise BBB integrity and thereby elicit neurological sequelae; IFN administration can restore barrier function and attenuate neuro-invasion. Third, flaviviral structural and non-structural proteins degrade pivotal molecules of the IFN cascade; IFN therapy is expected to upregulate the expression of these targets, thereby reversing virus-driven immunosuppression. Sparse but convergent clinical data further indicate that IFN alleviates selected symptoms in flavivirus-infected patients. Although definitive evidence remains scarce, IFN remains a highly promising candidate for antiviral intervention against flaviviruses.

## 8 Conclusion

In summary, the conflict between flaviviruses and our interferon system is a masterclass in biological warfare. These viruses have evolved a diverse toolkit to dismantle the host’s IFN response at nearly every turn-from the initial detection of the pathogen to the final execution of antiviral commands in the cell. This relentless evasion is fundamental to their ability to cause disease.

Nowhere is this battle more consequential than at the body’s vital barriers: the placenta and the blood-brain barrier (BBB). Here, interferons play a critical yet double-edged role. They are essential for controlling viral replication, but their response must be precisely calibrated. Too much or mistimed signaling can itself lead to damage, such as fetal growth restriction or a leaky BBB. Viruses, in turn, employ tactics like using infected cells as “Trojan horses” or directly breaking down cellular seals to cross these barriers, while IFNs struggle to maintain the integrity of these frontiers.

It is important to note that much of this detailed understanding comes from cellular models in the lab, which cannot fully mimic the complexity of a living organism. This gap underscores the need for more realistic animal models to confirm these mechanisms. Nevertheless, studies in genetically modified mice clearly prove that a functional interferon system is non-negotiable for protecting the central nervous system from viral invasion.

Looking ahead, this intricate knowledge opens promising therapeutic avenues. Boosting the interferon response-either by administering IFNs or using drugs that enhance their signaling-could help reinforce our natural barriers and suppress viruses. A key challenge will be finding the right therapeutic window, especially since virus-induced damage to barriers might, paradoxically, be exploited to deliver drugs. Ultimately, deciphering the complex dialogue between flaviviruses and our interferon system does more than explain how these pathogens make us sick. It lights the way toward smarter, immune-based strategies to prevent and treat the serious diseases they cause.

## Glossary

IFN: interferon
ISG: interferon-stimulated gene
PRR: pattern recognition receptor
JEV: Japanese encephalitis virus
WNV: West Nile virus
DENV: dengue virus
YFV: yellow fever virus
ZIKV: Zika virus
JE: Japanese encephalitis
JES: Japanese encephalitis complex
WNF: West Nile fever
WNND: West Nile neuroinvasive disease
DF: dengue fever
DHF: dengue hemorrhagic fever
DSS: dengue shock syndrome
GBS: Guillain-Barré syndrome
CZS: congenital Zika syndrome
CNS: central nervous system
KFDV: Kyasanur Forest disease virus
TBEV: Tick-borne encephalitis virus
TBE: tick-borne encephalitis
OHF: Omsk Hemorrhagic Fever
OHFV: Omsk Hemorrhagic Fever Virus
SLEV: Saint Louis encephalitis virus
SLE: Saint Louis encephalitis
+ssRNA: positive, single-stranded RNA
ORF: open reading frame
C: capsid
prM: precursor membrane
E: envelope
NC: nucleocapsid
ER: endoplasmic reticulum
RC: replication complex
VP: vesicle packet
RdRp: RNA-dependent RNA polymerase
ssRNA: single-stranded RNA
dsRNA: double-stranded RNA
TGN: trans-Golgi network
BBB: blood-brain barrier
PAMP: pathogen-associated molecular pattern
DAMP: damage-associated molecular pattern
TLR: Toll-like receptor
RLRs: retinoic acid-inducible gene-I (RIG-I)-like receptors
NLR: NOD-like receptor
cGAS: cyclic GMP-AMP synthase
IRF: interferon regulatory factor
NF-κB: nuclear factor-κB
MAPKs: mitogen-activated protein kinases
TBK1: TANK-binding kinase 1
IKK: inhibitory κB kinase
RIG-I: retinoic acid-inducible gene I
MDA5: melanoma differentiation-associated antigen 5
LGP2: Laboratory of Genetics and Physiology 2
CTD: C-terminal domain
CARD: caspase activation and recruitment domain
MAVS/IPS-1: mitochondrial antiviral signalling protein
mtDNA: mitochondrial DNA
cGAMP: cyclic GMP-AMP
ISGF3: interferon-stimulated gene factor 3
ISRE: IFN-stimulated response element
sfRNA: short fragment ncRNA
SOCS5: suppressor of cytokine signalling 5
DENV2: DENV serotype 2
PCSK9: proprotein convertase subtilisin/kexin type 9
CL: CARD-like
TM: transmembrane
PEPD: prolidase
pY-STAT1: STAT1 phosphorylation
ILK: integrin-linked kinase
VCT: villous cytotrophoblast
STB: syncytiotrophoblast
EVT: extravillous trophoblast
TGC: trophoblast giant cell
EV: extracellular vesicle
dpc: days post-coitus
IUGR: intrauterine growth restriction
HC: Hofbauer cell
CTB: cytotrophoblast
ECs: endothelial cells
AJ: adherens junction
TJs: tight junctions
JAMs: junctional adhesion molecules
NVU: neurovascular unit
MIF: migration inhibitory factor
MC: mast cell
BMEC: brain microvascular endothelial cell
hBMECs: human brain microvascular endothelial cells
EGFR: epidermal growth factor receptor
ERK: extracellular signal-regulated kinase
A129: IFN-α/β receptor-deficient mice
SOT: solid organ transplant
IVIG: intravenous immunoglobulin

## References

[B1] Aguirre S. Fernandez-Sesma A. (2017). Collateral damage during dengue virus infection: making sense of DNA by cGAS. J. Virol. 91 (14). 10.1128/JVI.01081-16 28446670 PMC5487551

[B2] Aguirre S. Maestre A. M. Pagni S. Patel J. R. Savage T. Gutman D. (2012). DENV inhibits type I IFN production in infected cells by cleaving human STING. PLoS Pathog. 8 (10), e1002934. 10.1371/journal.ppat.1002934 23055924 PMC3464218

[B3] Aguirre S. Luthra P. Sanchez-Aparicio M. T. Maestre A. M. Patel J. Lamothe F. (2017). Dengue virus NS2B protein targets cGAS for degradation and prevents mitochondrial DNA sensing during infection. Nat. Microbiol. 2, 17037. 10.1038/nmicrobiol.2017.37 28346446 PMC7457382

[B4] Airo A. M. Felix-Lopez A. Mancinelli V. Evseev D. Lopez-Orozco J. Shire K. (2022). Flavivirus capsid proteins inhibit the interferon response. Viruses 14 (5), 968. 10.3390/v14050968 35632712 PMC9146811

[B5] Alimonti J. B. Ribecco-Lutkiewicz M. Sodja C. Jezierski A. Stanimirovic D. B. Liu Q. (2018). Zika virus crosses an *in vitro* human blood brain barrier model. Fluids Barriers CNS 15 (1), 15. 10.1186/s12987-018-0100-y 29759080 PMC5952854

[B6] Alippe Y. Wang L. Coskun R. Muraro S. P. Zhao F. R. Elam-Noll M. (2024). Fetal MAVS and type I IFN signaling pathways control ZIKV infection in the placenta and maternal decidua. J. Exp. Med. 221 (9), e20240694. 10.1084/jem.20240694 39042188 PMC11270594

[B7] Ardakani R. Chauhan L. Piquet A. L. Tyler K. L. Pastula D. M. (2024). An overview of Saint Louis encephalitis. Neurohospitalist 14 (2), 230–231. 10.1177/19418744241228006 38666278 PMC11040620

[B8] Arumugasaamy N. Rock K. D. Kuo C. Y. Bale T. L. Fisher J. P. (2020). Microphysiological systems of the placental barrier. Adv. Drug Deliv. Rev. 161, 161–175. 10.1016/j.addr.2020.08.010 32858104 PMC10288517

[B9] Ashour J. Laurent-Rolle M. Shi P. Y. García-Sastre A. (2009). NS5 of dengue virus mediates STAT2 binding and degradation. J. Virol. 83 (11), 5408–5418. 10.1128/JVI.02188-08 19279106 PMC2681973

[B10] Azamor T. Cunha D. P. Nobre Pires K. S. Lira Tanabe E. L. Melgaço J. G. Vieira da Silva A. M. (2024). Decidual production of interferon lambda in response to ZIKV persistence: clinical evidence and *in vitro* modelling. Heliyon 10 (9), e30613. 10.1016/j.heliyon.2024.e30613 38737240 PMC11087979

[B11] Bayer A. Lennemann N. J. Ouyang Y. Bramley J. C. Morosky S. Marques E. T. D. A. (2016). Type III interferons produced by human placental trophoblasts confer protection against zika virus infection. Cell Host Microbe 19 (5), 705–712. 10.1016/j.chom.2016.03.008 27066743 PMC4866896

[B12] Bhardwaj U. Singh S. K. (2021). Zika virus NS1 suppresses VE-Cadherin and Claudin-5 via hsa-miR-101-3p in human brain microvascular endothelial cells. Mol. Neurobiol. 58 (12), 6290–6303. 10.1007/s12035-021-02548-x 34487317

[B13] Bhardwaj U. Singh S. K. (2023). Zika virus NS1 suppresses VE-cadherin via hsa-miR-29b-3p/DNMT3b/MMP-9 pathway in human brain microvascular endothelial cells. Cell Signal 106, 110659. 10.1016/j.cellsig.2023.110659 36948479

[B14] Borden E. C. Sen G. C. Uze G. Silverman R. H. Ransohoff R. M. Foster G. R. (2007). Interferons at age 50: past, current and future impact on biomedicine. Nat. Rev. Drug Discov. 6 (12), 975–990. 10.1038/nrd2422 18049472 PMC7097588

[B15] Campbell G. L. Hills S. L. Fischer M. Jacobson J. A. Hoke C. H. Hombach J. M. (2011). Estimated global incidence of Japanese encephalitis: a systematic review. Bull. World Health Organ. 89 (10), 74a–74e. 10.2471/BLT.10.085233 22084515 PMC3209971

[B16] Campbell H. K. Maiers J. L. Demali K. A. (2017). Interplay between tight junctions & adherens junctions. Exp. Cell Res. 358 (1), 39–44. 10.1016/j.yexcr.2017.03.061 28372972 PMC5544570

[B17] Cao X. (2016). Self-regulation and cross-regulation of pattern-recognition receptor signalling in health and disease. Nat. Rev. Immunol. 16 (1), 35–50. 10.1038/nri.2015.8 26711677

[B18] Cao-Lormeau V. M. Blake A. Mons S. Lastère S. Roche C. Vanhomwegen J. (2016). Guillain-barré syndrome outbreak associated with zika virus infection in French Polynesia: a case-control study. Lancet 387 (10027), 1531–1539. 10.1016/S0140-6736(16)00562-6 26948433 PMC5444521

[B19] Carty M. Guy C. Bowie A. G. (2021). Detection of viral infections by innate immunity. Biochem. Pharmacol. 183, 114316. 10.1016/j.bcp.2020.114316 33152343

[B20] Casazza R. L. Philip D. T. Lazear H. M. (2022). Interferon lambda signals in maternal tissues to exert protective and pathogenic effects in a gestational stage-dependent manner. mBio 13 (3), e0385721. 10.1128/mbio.03857-21 35471083 PMC9239100

[B21] Chakraborty S. Andrade F. C. D. Ghosh S. Uelmen J. Ruiz M. O. (2019). Historical expansion of kyasanur forest disease in India from 1957 to 2017: a retrospective analysis. Geohealth 3 (2), 44–55. 10.1029/2018GH000164 32159030 PMC7007137

[B22] Chang R. Y. Hsu T. W. Chen Y. L. Liu S. F. Tsai Y. J. Lin Y. T. (2013). Japanese encephalitis virus non-coding RNA inhibits activation of interferon by blocking nuclear translocation of interferon regulatory factor 3. Vet. Microbiol. 166 (1-2), 11–21. 10.1016/j.vetmic.2013.04.026 23755934

[B23] Chapagain S. Pal Singh P. Le K. Safronetz D. Wood H. Karniychuk U. (2022). Japanese encephalitis virus persists in the human reproductive epithelium and porcine reproductive tissues. PLoS Negl. Trop. Dis. 16 (7), e0010656. 10.1371/journal.pntd.0010656 35905074 PMC9337681

[B24] Chen J. Liang Y. Yi P. Xu L. Hawkins H. K. Rossi S. L. (2017). Outcomes of congenital zika disease depend on timing of infection and maternal-fetal interferon action. Cell Rep. 21 (6), 1588–1599. 10.1016/j.celrep.2017.10.059 29117563 PMC5726784

[B25] Chen J. Yang Y. F. Yang Y. Zou P. Chen J. He Y. (2018). AXL promotes zika virus infection in astrocytes by antagonizing type I interferon signalling. Nat. Microbiol. 3 (3), 302–309. 10.1038/s41564-017-0092-4 29379210

[B26] Chen W. Li Y. Yu X. Wang Z. Wang W. Rao M. (2023). Zika virus non-structural protein 4B interacts with DHCR7 to facilitate viral infection. Virol. Sin. 38 (1), 23–33. 10.1016/j.virs.2022.09.009 36182074 PMC10006206

[B27] Chiu C. F. Chu L. W. Liao I. C. Simanjuntak Y. Lin Y. L. Juan C. C. (2020). The mechanism of the zika virus crossing the placental barrier and the blood-brain barrier. Front. Microbiol. 11, 214. 10.3389/fmicb.2020.00214 32153526 PMC7044130

[B28] Chow K. T. Gale M. JR (2015). SnapShot: Interferon signaling. Cell 163 (7), 1808-.e1. 10.1016/j.cell.2015.12.008 26687364

[B29] Chugh P. Soni S. Ghanghas N. Kumar S. Mohan H. (2025). Comprehensive insights into Japanese encephalitis virus: from molecular characterization to advanced detection and vaccine strategies. Antivir. Res. 243, 106268. 10.1016/j.antiviral.2025.106268 40907709

[B30] Cook B. W. Cutts T. A. Court D. A. Theriault S. (2012). The generation of a reverse genetics system for kyasanur forest disease virus and the ability to antagonize the induction of the antiviral state *in vitro* . Virus Res. 163 (2), 431–438. 10.1016/j.virusres.2011.11.002 22100401

[B31] Cui F. He F. Huang X. Tian L. Li S. Liang C. (2022). Dengue and dengue virus in Guangdong, China, 1978-2017: epidemiology, seroprevalence, evolution, and policies. Front. Med. (Lausanne) 9, 797674. 10.3389/fmed.2022.797674 35386910 PMC8979027

[B32] Dalrymple N. A. Cimica V. Mackow E. R. (2015). Dengue virus NS proteins inhibit RIG-I/MAVS signaling by blocking TBK1/IRF3 phosphorylation: dengue virus serotype 1 NS4A is a unique interferon-regulating virulence determinant. mBio 6 (3), e00553. 10.1128/mBio.00553-15 25968648 PMC4436066

[B33] Davis L. E. Debiasi R. Goade D. E. Haaland K. Y. Harrington J. A. Harnar J. B. (2006). West nile virus neuroinvasive disease. Ann. Neurol. 60 (3), 286–300. 10.1002/ana.20959 16983682

[B34] De Araújo T. V. B. Rodrigues L. C. De Alencar Ximenes R. A. de Barros Miranda-Filho D. Montarroyos U. R. de Melo A. P. L. (2016). Association between zika virus infection and microcephaly in Brazil, January to may, 2016: preliminary report of a case-control study. Lancet Infect. Dis. 16 (12), 1356–1363. 10.1016/S1473-3099(16)30318-8 27641777 PMC7617035

[B35] De Weerd N. A. Vivian J. P. Nguyen T. K. Mangan N. E. Gould J. A. Braniff S. J. (2013). Structural basis of a unique interferon-β signaling axis mediated via the receptor IFNAR1. Nat. Immunol. 14 (9), 901–907. 10.1038/ni.2667 23872679

[B36] Diani E. Cecchetto R. Tonon E. Mantoan M. Lotti V. Lagni A. (2025). Omsk hemorrhagic fever virus: a comprehensive review from epidemiology to diagnosis and treatment. Microorganisms 13 (2), 426. 10.3390/microorganisms13020426 40005791 PMC11858464

[B37] Dick G. W. Kitchen S. F. Haddow A. J. (1952). Zika virus. I. Isolations and serological specificity. Trans. R. Soc. Trop. Med. Hyg. 46 (5), 509–520. 10.1016/0035-9203(52)90042-4 12995440

[B38] Ding Q. Gaska J. M. Douam F. Wei L. Kim D. Balev M. (2018). Species-specific disruption of STING-Dependent antiviral cellular defenses by the zika virus NS2B3 protease. Proc. Natl. Acad. Sci. U. S. A. 115 (27), E6310. 10.1073/pnas.1803406115 29915078 PMC6142274

[B39] Ding J. Aldo P. Roberts C. M. Stabach P. Liu H. You Y. (2021). Placenta-derived interferon-stimulated gene 20 controls ZIKA virus infection. EMBO Rep. 22 (10), e52450. 10.15252/embr.202152450 34405956 PMC8490983

[B40] Doldan P. Dai J. Metz-Zumaran C. Patton J. T. Stanifer M. L. Boulant S. (2022). Type III and not type I interferons efficiently prevent the spread of rotavirus in human intestinal epithelial cells. J. Virol. 96 (17), e0070622. 10.1128/jvi.00706-22 36000839 PMC9472630

[B41] Donnelly R. P. Kotenko S. V. (2010). Interferon-lambda: a new addition to an old family. J. Interferon Cytokine Res. 30 (8), 555–564. 10.1089/jir.2010.0078 20712453 PMC2925029

[B42] Dowall S. D. Graham V. A. Rayner E. Atkinson B. Hall G. Watson R. J. (2016). A susceptible mouse model for zika virus infection. PLoS Negl. Trop. Dis. 10 (5), e0004658. 10.1371/journal.pntd.0004658 27149521 PMC4858159

[B43] Duffy M. R. Chen T. H. Hancock W. T. Powers A. M. Kool J. L. Lanciotti R. S. (2009). Zika virus outbreak on Yap island, Federated States of Micronesia. N. Engl. J. Med. 360 (24), 2536–2543. 10.1056/NEJMoa0805715 19516034

[B44] Erdő F. Denes L. De Lange E. (2017). Age-associated physiological and pathological changes at the blood-brain barrier: a review. J. Cereb. Blood Flow. Metab. 37 (1), 4–24. 10.1177/0271678X16679420 27837191 PMC5363756

[B45] Espada C. E. Da Rocha E. L. Ricciardi-Jorge T. Dos Santos A. A. Soares Z. G. Malaquias G. (2024). ISG15/USP18/STAT2 is a molecular hub regulating IFN I-mediated control of dengue and zika virus replication. Front. Immunol. 15, 1331731. 10.3389/fimmu.2024.1331731 38384473 PMC10879325

[B46] Evans J. D. Crown R. A. Sohn J. A. Seeger C. (2011). West nile virus infection induces depletion of IFNAR1 protein levels. Viral Immunol. 24 (4), 253–263. 10.1089/vim.2010.0126 21830897 PMC3154464

[B47] Fanunza E. Carletti F. Quartu M. Grandi N. Ermellino L. Milia J. (2021a). Zika virus NS2A inhibits interferon signaling by degradation of STAT1 and STAT2. Virulence 12 (1), 1580–1596. 10.1080/21505594.2021.1935613 34338586 PMC8331042

[B48] Fanunza E. Grandi N. Quartu M. Carletti F. Ermellino L. Milia J. (2021b). INMI1 zika virus NS4B antagonizes the interferon signaling by suppressing STAT1 phosphorylation. Viruses 13 (12), 2448. 10.3390/v13122448 34960717 PMC8705506

[B49] Fendereski M. Neupane B. Nazneen F. Bai F. Guo Y. L. (2022). Mouse trophoblast cells can provide IFN-based antiviral protection to embryonic stem cells via paracrine signaling. J. Immunol. 208 (12), 2761–2770. 10.4049/jimmunol.2100679 35649628 PMC9308691

[B50] Feng T. Sun T. Li G. Pan W. Wang K. Dai J. (2017). DEAD-box helicase DDX25 is a negative regulator of type I interferon pathway and facilitates RNA virus infection. Front. Cell Infect. Microbiol. 7, 356. 10.3389/fcimb.2017.00356 28824886 PMC5543031

[B51] Fikatas A. Dehairs J. Noppen S. Doijen J. Vanderhoydonc F. Meyen E. (2021). Deciphering the role of extracellular vesicles derived from ZIKV-infected hcMEC/D3 cells on the blood-brain barrier system. Viruses 13 (12), 2363. 10.3390/v13122363 34960632 PMC8708812

[B52] Fisher R. Lustig Y. Sklan E. H. Schwartz E. (2023). The role of NS1 protein in the diagnosis of flavivirus infections. Viruses 15 (2), 572. 10.3390/v15020572 36851784 PMC9963814

[B53] Gan E. S. Tan H. C. Le D. H. T. Huynh T. T. Wills B. Seidah N. G. (2020). Dengue virus induces PCSK9 expression to alter antiviral responses and disease outcomes. J. Clin. Invest. 130 (10), 5223–5234. 10.1172/JCI137536 32644974 PMC7524462

[B54] Garcia-Oliveira G. F. Guimarães A. Moreira G. D. Costa T. A. Arruda M. S. de Mello É. M. (2023). YELLOW ALERT: persistent yellow fever virus circulation among non-human Primates in urban areas of Minas Gerais state, Brazil (2021-2023). Viruses 16 (1), 31. 10.3390/v16010031 38257732 PMC10818614

[B55] Garske T. Van Kerkhove M. D. Yactayo S. Ronveaux O. Lewis R. F. Staples J. E. (2014). Yellow fever in Africa: estimating the burden of disease and impact of mass vaccination from outbreak and serological data. PLoS Med. 11 (5), e1001638. 10.1371/journal.pmed.1001638 24800812 PMC4011853

[B56] Gewaid H. Bowie A. G. (2024). Regulation of type I and type III interferon induction in response to pathogen sensing. Curr. Opin. Immunol. 87, 102424. 10.1016/j.coi.2024.102424 38761566

[B57] Giesen C. Herrador Z. Fernandez-Martinez B. Figuerola J. Gangoso L. Vazquez A. (2023). A systematic review of environmental factors related to WNV circulation in European and mediterranean countries. One Health 16, 100478. 10.1016/j.onehlt.2022.100478 37363246 PMC10288031

[B58] Grant A. Ponia S. S. Tripathi S. Balasubramaniam V. Miorin L. Sourisseau M. (2016). Zika virus targets human STAT2 to inhibit type I interferon signaling. Cell Host Microbe 19 (6), 882–890. 10.1016/j.chom.2016.05.009 27212660 PMC4900918

[B59] Gupta N. Wilson W. Neumayr A. Saravu K. (2022). Kyasanur forest disease: a state-of-the-art review. Qjm 115 (6), 351–358. 10.1093/qjmed/hcaa310 33196834

[B60] Gürtler C. Bowie A. G. (2013). Innate immune detection of microbial nucleic acids. Trends Microbiol. 21 (8), 413–420. 10.1016/j.tim.2013.04.004 23726320 PMC3735846

[B61] Hartmann C. A. Vikram H. R. Seville M. T. Orenstein R. Kusne S. Blair J. E. (2017). Neuroinvasive St. Louis encephalitis virus infection in solid organ transplant recipients. Am. J. Transpl. 17 (8), 2200–2206. 10.1111/ajt.14336 28452107

[B62] Hartsock A. Nelson W. J. (2008). Adherens and tight junctions: structure, function and connections to the actin cytoskeleton. Biochim. Biophys. Acta 1778 (3), 660–669. 10.1016/j.bbamem.2007.07.012 17854762 PMC2682436

[B63] Hasan M. N. Khalil I. Chowdhury M. A. B. Rahman M. Asaduzzaman M. Billah M. (2024). Two decades of endemic dengue in Bangladesh (2000-2022): trends, seasonality, and impact of temperature and rainfall patterns on transmission dynamics. J. Med. Entomol. 61 (2), 345–353. 10.1093/jme/tjae001 38253990 PMC10936167

[B64] Hazra B. Kumawat K. L. Basu A. (2017). The host microRNA miR-301a blocks the IRF1-mediated neuronal innate immune response to Japanese encephalitis virus infection. Sci. Signal 10 (466), eaaf5185. 10.1126/scisignal.aaf5185 28196914

[B65] He Z. Zhu X. Wen W. Yuan J. Hu Y. Chen J. (2016). Dengue virus subverts host innate immunity by targeting adaptor protein MAVS. J. Virol. 90 (16), 7219–7230. 10.1128/JVI.00221-16 27252539 PMC4984625

[B66] Hennessey M. Fischer M. Staples J. E. (2016). Zika virus spreads to new areas - region of the americas, may 2015-January 2016. MMWR Morb. Mortal. Wkly. Rep. 65 (3), 55–58. 10.15585/mmwr.mm6503e1 26820163

[B67] Hertzog J. Dias Junior A. G. Rigby R. E. Donald C. L. Mayer A. Sezgin E. (2018). Infection with a Brazilian isolate of zika virus generates RIG-I stimulatory RNA and the viral NS5 protein blocks type I IFN induction and signaling. Eur. J. Immunol. 48 (7), 1120–1136. 10.1002/eji.201847483 29572905 PMC6055886

[B68] Honein M. A. Dawson A. L. Petersen E. E. Jones A. M. Lee E. H. Yazdy M. M. (2017). Birth defects among fetuses and infants of US women with evidence of possible zika virus infection during pregnancy. Jama 317 (1), 59–68. 10.1001/jama.2016.19006 27960197

[B69] Horga A. Teixeira M. M. Borthakur S. Lynch S. Chin J. Ishak L. (2025). Safety, pharmacokinetics, and activity of AT-752, a novel nucleotide prodrug with pan-serotype activity against dengue virus: a phase 2, randomized, double-blind study. Am. J. Trop. Med. Hyg. 113 (3), 498–507. 10.4269/ajtmh.24-0696 40639366 PMC12410160

[B70] Hsieh J. T. Rathore A. P. S. Soundarajan G. St John A. L. (2019). Japanese encephalitis virus neuropenetrance is driven by mast cell chymase. Nat. Commun. 10 (1), 706. 10.1038/s41467-019-08641-z 30742008 PMC6370868

[B71] Hu Y. Dong X. He Z. Wu Y. Zhang S. Lin J. (2019). Zika virus antagonizes interferon response in patients and disrupts RIG-I-MAVS interaction through its CARD-TM domains. Cell Biosci. 9, 46. 10.1186/s13578-019-0308-9 31183075 PMC6555941

[B72] Hu T. Wu Z. Wu S. Chen S. Cheng A. (2021). The key amino acids of E protein involved in early flavivirus infection: viral entry. Virol. J. 18 (1), 136. 10.1186/s12985-021-01611-2 34217298 PMC8254458

[B73] Hu H. Feng Y. He M. L. (2023). Targeting type I interferon induction and signaling: how zika virus escapes from host innate immunity. Int. J. Biol. Sci. 19 (10), 3015–3028. 10.7150/ijbs.83056 37416780 PMC10321277

[B74] Isaacs A. Lindenmann J. (1957). Virus interference. I. The interferon. Proc. R. Soc. Lond B Biol. Sci. 147 (927), 258–267. 10.1098/rspb.1957.0048 13465720

[B75] Jablunovsky A. Jose J. (2024). The dynamic landscape of capsid proteins and viral RNA interactions in flavivirus genome packaging and virus assembly. Pathogens 13 (2), 120. 10.3390/pathogens13020120 38392858 PMC10893219

[B76] Jagger B. W. Miner J. J. Cao B. Arora N. Smith A. M. Kovacs A. (2017). Gestational stage and IFN-λ signaling regulate ZIKV infection in utero. Cell Host Microbe 22 (3), 366–376. 10.1016/j.chom.2017.08.012 28910635 PMC5647680

[B77] Julander J. G. Winger Q. A. Rickords L. F. Shi P. Y. Tilgner M. Binduga-Gajewska I. (2006). West nile virus infection of the placenta. Virology 347 (1), 175–182. 10.1016/j.virol.2005.11.040 16406457

[B78] Kao Y. S. Wang L. C. Chang P. C. Lin H. M. Lin Y. S. Yu C. Y. (2023). Negative regulation of type I interferon signaling by integrin-linked kinase permits dengue virus replication. PLoS Pathog. 19 (3), e1011241. 10.1371/journal.ppat.1011241 36930690 PMC10057834

[B79] Kasule S. N. Gupta S. Patron R. L. Grill M. F. Vikram H. R. (2023). Neuroinvasive west nile virus infection in solid organ transplant recipients. Transpl. Infect. Dis. 25 (1), e14004. 10.1111/tid.14004 36573623

[B80] Kaur G. Pant P. Bhagat R. Seth P. (2023). Zika virus E protein modulates functions of human brain microvascular endothelial cells and astrocytes: implications on blood-brain barrier properties. Front. Cell Neurosci. 17, 1173120. 10.3389/fncel.2023.1173120 37545876 PMC10399241

[B81] Kawai T. Akira S. (2010). The role of pattern-recognition receptors in innate immunity: update on toll-like receptors. Nat. Immunol. 11 (5), 373–384. 10.1038/ni.1863 20404851

[B82] Kim N. E. Song Y. J. (2022). Coordinated regulation of interferon and inflammasome signaling pathways by SARS-CoV-2 proteins. J. Microbiol. 60 (3), 300–307. 10.1007/s12275-022-1502-8 35089584 PMC8795727

[B83] Knyazhanskaya E. Morais M. C. Choi K. H. (2021). Flavivirus enzymes and their inhibitors. Enzymes 49, 265–303. 10.1016/bs.enz.2021.07.006 34696835 PMC8717743

[B84] Kularatne S. A. Dalugama C. (2022). Dengue infection: global importance, immunopathology and management. Clin. Med. (Lond) 22 (1), 9–13. 10.7861/clinmed.2021-0791 35078789 PMC8813012

[B85] Kundu K. Dutta K. Nazmi A. Basu A. (2013). Japanese encephalitis virus infection modulates the expression of suppressors of cytokine signaling (SOCS) in macrophages: implications for the hosts' innate immune response. Cell Immunol. 285 (1-2), 100–110. 10.1016/j.cellimm.2013.09.005 24140964

[B86] Kwasnik M. Rola J. Rozek W. (2023). Tick-borne encephalitis-review of the current status. J. Clin. Med. 12 (20), 6603. 10.3390/jcm12206603 37892741 PMC10607749

[B87] Labib B. A. Chigbu D. I. (2022). Pathogenesis and manifestations of zika virus-associated ocular diseases. Trop. Med. Infect. Dis. 7 (6), 106. 10.3390/tropicalmed7060106 35736984 PMC9229560

[B88] Laurent-Rolle M. Boer E. F. Lubick K. J. Wolfinbarger J. B. Carmody A. B. Rockx B. (2010). The NS5 protein of the virulent west nile virus NY99 strain is a potent antagonist of type I interferon-mediated JAK-STAT signaling. J. Virol. 84 (7), 3503–3515. 10.1128/JVI.01161-09 20106931 PMC2838099

[B89] Lazear H. M. Daniels B. P. Pinto A. K. Huang A. C. Vick S. C. Doyle S. E. (2015). Interferon-λ restricts west nile virus neuroinvasion by tightening the blood-brain barrier. Sci. Transl. Med. 7 (284), 284ra59. 10.1126/scitranslmed.aaa4304 25904743 PMC4435724

[B90] Lazear H. M. Schoggins J. W. Diamond M. S. (2019). Shared and distinct functions of type I and type III interferons. Immunity 50 (4), 907–923. 10.1016/j.immuni.2019.03.025 30995506 PMC6839410

[B91] Levkovitz R. Zaretsky U. Gordon Z. Jaffa A. J. Elad D. (2013). *In vitro* simulation of placental transport: part I. Biological model of the placental barrier. Placenta 34 (8), 699–707. 10.1016/j.placenta.2013.03.014 23764139

[B92] Li F. Wang Y. Yu L. Cao S. Wang K. Yuan J. (2015). Viral infection of the central nervous system and neuroinflammation precede blood-brain barrier disruption during Japanese encephalitis virus infection. J. Virol. 89 (10), 5602–5614. 10.1128/JVI.00143-15 25762733 PMC4442524

[B93] Li A. Wang W. Wang Y. Chen K. Xiao F. Hu D. (2020). NS5 conservative site is required for zika virus to restrict the RIG-I signaling. Front. Immunol. 11, 51. 10.3389/fimmu.2020.00051 32117232 PMC7033454

[B94] Li Y. Li Z. Zou H. Zhou P. Huo Y. Fan Y. (2024). A conserved methyltransferase active site residue of zika virus NS5 is required for the restriction of STING activation and interferon expression. J. Gen. Virol. 105 (2). 10.1099/jgv.0.001954 38299799

[B95] Lin R. J. Chang B. L. Yu H. P. Liao C. L. Lin Y. L. (2006). Blocking of interferon-induced jak-stat signaling by Japanese encephalitis virus NS5 through a protein tyrosine phosphatase-mediated mechanism. J. Virol. 80 (12), 5908–5918. 10.1128/JVI.02714-05 16731929 PMC1472572

[B96] Lin C. W. Cheng C. W. Yang T. C. Li S. W. Cheng M. H. Wan L. (2008). Interferon antagonist function of Japanese encephalitis virus NS4A and its interaction with DEAD-box RNA helicase DDX42. Virus Res. 137 (1), 49–55. 10.1016/j.virusres.2008.05.015 18588927

[B97] Lin S. Yang S. He J. Guest J. D. Ma Z. Yang L. (2019). Zika virus NS5 protein antagonizes type I interferon production via blocking TBK1 activation. Virology 527, 180–187. 10.1016/j.virol.2018.11.009 30530224 PMC6340140

[B98] Low J. G. Ng J. H. J. Ong E. Z. Kalimuddin S. Wijaya L. Chan Y. F. Z. (2020). Phase 1 trial of a therapeutic anti-yellow fever virus human antibody. N. Engl. J. Med. 383 (5), 452–459. 10.1056/NEJMoa2000226 32726531

[B99] Lu J. Gullett J. M. Kanneganti T. D. (2022). Filoviruses: innate immunity, inflammatory cell death, and cytokines. Pathogens 11 (12), 1400. 10.3390/pathogens11121400 36558734 PMC9785368

[B100] Lubick K. J. Robertson S. J. Mcnally K. L. Freedman B. A. Rasmussen A. L. Taylor R. T. (2015). Flavivirus antagonism of type I interferon signaling reveals prolidase as a regulator of IFNAR1 surface expression. Cell Host Microbe 18 (1), 61–74. 10.1016/j.chom.2015.06.007 26159719 PMC4505794

[B101] Lundberg R. Melén K. Westenius V. Jiang M. Österlund P. Khan H. (2019). Zika virus non-structural protein NS5 inhibits the RIG-I pathway and interferon lambda 1 promoter activation by targeting IKK epsilon. Viruses 11 (11). 10.3390/v11111024 31690057 PMC6893776

[B102] Lynn M. K. Aquino M. S. R. Self S. C. W. Kanyangarara M. Campbell B. A. Nolan M. S. (2023). TORCH congenital syndrome infections in central america's northern triangle. Microorganisms 11 (2), 257. 10.3390/microorganisms11020257 36838223 PMC9964893

[B103] Ma J. Ketkar H. Geng T. Lo E. Wang L. Xi J. (2018). Zika virus non-structural protein 4A blocks the RLR-MAVS signaling. Front. Microbiol. 9, 1350. 10.3389/fmicb.2018.01350 29988497 PMC6026624

[B104] Manocha G. D. Mishra R. Sharma N. Kumawat K. L. Basu A. Singh S. K. (2014). Regulatory role of TRIM21 in the type-I interferon pathway in Japanese encephalitis virus-infected human microglial cells. J. Neuroinflammation 11, 24. 10.1186/1742-2094-11-24 24485101 PMC3922089

[B105] Mansfield K. L. Johnson N. Phipps L. P. Stephenson J. R. Fooks A. R. Solomon T. (2009). Tick-borne encephalitis virus - a review of an emerging zoonosis. J. Gen. Virol. 90 (Pt 8), 1781–1794. 10.1099/vir.0.011437-0 19420159

[B106] Marrs C. Olson G. Saade G. Hankins G. Wen T. Patel J. (2016). Zika virus and pregnancy: a review of the literature and clinical considerations. Am. J. Perinatol. 33 (7), 625–639. 10.1055/s-0036-1580089 26939047 PMC5214529

[B107] Martín-Acebes M. A. Saiz J. C. (2012). West nile virus: a re-emerging pathogen revisited. World J. Virol. 1 (2), 51–70. 10.5501/wjv.v1.i2.51 24175211 PMC3782267

[B108] Mattar S. Ojeda C. Arboleda J. Arrieta G. Bosch I. Botia I. (2017). Case report: microcephaly associated with zika virus infection, Colombia. BMC Infect. Dis. 17 (1), 423. 10.1186/s12879-017-2522-6 28610628 PMC5470308

[B109] Mehla R. Kumar S. R. Yadav P. Barde P. V. Yergolkar P. N. Erickson B. R. (2009). Recent ancestry of kyasanur forest disease virus. Emerg. Infect. Dis. 15 (9), 1431–1437. 10.3201/eid1509.080759 19788811 PMC2819879

[B110] Meier K. C. Gardner C. L. Khoretonenko M. V. Klimstra W. B. Ryman K. D. (2009). A mouse model for studying viscerotropic disease caused by yellow fever virus infection. PLoS Pathog. 5 (10), e1000614. 10.1371/journal.ppat.1000614 19816561 PMC2749449

[B111] Meneses J. D. A. Ishigami A. C. De Mello L. M. de Albuquerque L. L. de Brito C. A. A. Cordeiro M. T. (2017). Lessons learned at the epicenter of Brazil's congenital zika epidemic: evidence from 87 confirmed cases. Clin. Infect. Dis. 64 (10), 1302–1308. 10.1093/cid/cix166 28329257

[B112] Mladinich M. C. Schwedes J. Mackow E. R. (2017). Zika virus persistently infects and is basolaterally released from primary human brain microvascular endothelial cells. mBio 8 (4). 10.1128/mBio.00952-17 28698279 PMC5513708

[B113] Morita E. Suzuki Y. (2021). Membrane-associated flavivirus replication complex-its organization and regulation. Viruses 13 (6), 1060. 10.3390/v13061060 34205058 PMC8228428

[B114] Morrison J. Laurent-Rolle M. Maestre A. M. Rajsbaum R. Pisanelli G. Simon V. (2013). Dengue virus co-opts UBR4 to degrade STAT2 and antagonize type I interferon signaling. PLoS Pathog. 9 (3), e1003265. 10.1371/journal.ppat.1003265 23555265 PMC3610674

[B115] Mulvey P. Duong V. Boyer S. Burgess G. Williams D. T. Dussart P. (2021). The ecology and evolution of Japanese encephalitis virus. Pathogens 10 (12), 1534. 10.3390/pathogens10121534 34959489 PMC8704921

[B116] Murphy B. R. Whitehead S. S. (2011). Immune response to dengue virus and prospects for a vaccine. Annu. Rev. Immunol. 29, 587–619. 10.1146/annurev-immunol-031210-101315 21219187

[B117] N S. Kandi V. G S. R. Ca J. A H. As A. (2024). Kyasanur forest disease: a comprehensive review. Cureus 16 (7), e65228. 10.7759/cureus.65228 39184677 PMC11343324

[B118] Ngueyen T. T. N. Kim S. J. Lee J. Y. Myoung J. (2019). Zika virus proteins NS2A and NS4A are major antagonists that reduce IFN-β promoter activity induced by the MDA5/RIG-I signaling pathway. J. Microbiol. Biotechnol. 29 (10), 1665–1674. 10.4014/jmb.1909.09017 31581385

[B119] Nie Y. Deng D. Mou L. Long Q. Chen J. Wu J. (2023). Dengue virus 2 NS2B targets MAVS and IKKε to evade the antiviral innate immune response. J. Microbiol. Biotechnol. 33 (5), 600–606. 10.4014/jmb.2210.10006 36788451 PMC10236164

[B120] Odendall C. Kagan J. C. (2015). The unique regulation and functions of type III interferons in antiviral immunity. Curr. Opin. Virol. 12, 47–52. 10.1016/j.coviro.2015.02.003 25771505 PMC4470718

[B121] Onomoto K. Onoguchi K. Yoneyama M. (2021). Regulation of RIG-I-like receptor-mediated signaling: interaction between host and viral factors. Cell Mol. Immunol. 18 (3), 539–555. 10.1038/s41423-020-00602-7 33462384 PMC7812568

[B122] Pan Y. Cai W. Cheng A. Wang M. Yin Z. Jia R. (2022). Flaviviruses: innate immunity, inflammasome activation, inflammatory cell death, and cytokines. Front. Immunol. 13, 829433. 10.3389/fimmu.2022.829433 35154151 PMC8835115

[B123] Park A. Iwasaki A. (2020). Type I and type III interferons - induction, signaling, evasion, and application to combat COVID-19. Cell Host Microbe 27 (6), 870–878. 10.1016/j.chom.2020.05.008 32464097 PMC7255347

[B124] Paul A. M. Acharya D. Le L. Wang P. Stokic D. S. Leis A. A. (2016). TLR8 couples SOCS-1 and restrains TLR7-Mediated antiviral immunity, exacerbating west nile virus infection in mice. J. Immunol. 197 (11), 4425–4435. 10.4049/jimmunol.1600902 27798161 PMC5123688

[B125] Petersen L. R. Brault A. C. Nasci R. S. (2013). West nile virus: review of the literature. Jama 310 (3), 308–315. 10.1001/jama.2013.8042 23860989 PMC4563989

[B126] Pierson T. C. Diamond M. S. (2020). The continued threat of emerging flaviviruses. Nat. Microbiol. 5 (6), 796–812. 10.1038/s41564-020-0714-0 32367055 PMC7696730

[B127] Plociennikowska A. Frankish J. Moraes T. Del Prete D. Kahnt F. Acuna C. (2021). TLR3 activation by zika virus stimulates inflammatory cytokine production which dampens the antiviral response induced by RIG-I-like receptors. J. Virol. 95 (10), e01050-20. 10.1128/JVI.01050-20 33658344 PMC8139665

[B128] Postler T. S. Beer M. Blitvich B. J. Bukh J. de Lamballerie X. Drexler J. F. (2023). Renaming of the genus flavivirus to orthoflavivirus and extension of binomial species names within the family flaviviridae. Arch. Virol. 168 (9), 224. 10.1007/s00705-023-05835-1 37561168

[B129] Profaci C. P. Munji R. N. Pulido R. S. Daneman R. (2020). The blood-brain barrier in health and disease: important unanswered questions. J. Exp. Med. 217 (4). 10.1084/jem.20190062 32211826 PMC7144528

[B130] Quicke K. M. Bowen J. R. Johnson E. L. McDonald C. E. MA H. O'Neal J. T. (2016). Zika virus infects human placental macrophages. Cell Host Microbe 20 (1), 83–90. 10.1016/j.chom.2016.05.015 27247001 PMC5166429

[B131] Rahal J. J. Anderson J. Rosenberg C. Reagan T. Thompson L. L. (2004). Effect of interferon-alpha2b therapy on St. Louis viral meningoencephalitis: clinical and laboratory results of a pilot study. J. Infect. Dis. 190 (6), 1084–1087. 10.1086/423325 15319857

[B132] Relich R. F. Loeffelholz M. (2017). Zika virus. Clin. Lab. Med. 37 (2), 253–267. 10.1016/j.cll.2017.01.002 28457349

[B133] Ren W. Fu C. Zhang Y. Ju X. Jiang X. Song J. (2024). Zika virus NS5 protein inhibits type I interferon signaling via CRL3 E3 ubiquitin ligase-mediated degradation of STAT2. Proc. Natl. Acad. Sci. U. S. A. 121 (34), e2403235121. 10.1073/pnas.2403235121 39145933 PMC11348293

[B134] Reyes V. A. Kane S. Glynn-Robinson A. (2025). Emergence of locally acquired Japanese encephalitis virus in Australia, January 2021-June 2022: a national case series. Commun. Dis. Intell., 49. 10.33321/cdi.2025.49.005 39837615

[B135] Roby J. A. Setoh Y. X. Hall R. A. Khromykh A. A. (2015). Post-translational regulation and modifications of flavivirus structural proteins. J. Gen. Virol. 96 (Pt 7), 1551–1569. 10.1099/vir.0.000097 25711963

[B136] Rocha R. F. Del Sarto J. L. Gomes G. F. Gonçalves M. P. Rachid M. A. Smetana J. H. C. (2021). Type I interferons are essential while type II interferon is dispensable for protection against St. Louis encephalitis virus infection in the mouse brain. Virulence 12 (1), 244–259. 10.1080/21505594.2020.1869392 33410731 PMC7808420

[B137] Rodhain F. (2022). Yellow fever: a brief history of a tropical virosis. Presse Med. 51 (3), 104132. 10.1016/j.lpm.2022.104132 35667600

[B138] Roe K. Kumar M. Lum S. Orillo B. Nerurkar V. R. Verma S. (2012). West nile virus-induced disruption of the blood-brain barrier in mice is characterized by the degradation of the junctional complex proteins and increase in multiple matrix metalloproteinases. J. Gen. Virol. 93 (Pt 6), 1193–1203. 10.1099/vir.0.040899-0 22398316 PMC3755517

[B139] Roy S. K. Bhattacharjee S. (2021). Dengue virus: epidemiology, biology, and disease aetiology. Can. J. Microbiol. 67 (10), 687–702. 10.1139/cjm-2020-0572 34171205

[B140] Rust L. N. Ricciardi M. J. Lutz S. S. Yusova S. Louw J. J. Yrizarry-Medina A. (2025). Prophylactic and therapeutic neutralizing monoclonal antibody treatment prevents lethal yellow fever infection. JCI Insight 10 (16), e191665. 10.1172/jci.insight.191665 40663406 PMC12416903

[B141] Saiz J. C. Martín-Acebes M. A. Blázquez A. B. Escribano-Romero E. Poderoso T. Jiménez de Oya N. (2021). Pathogenicity and virulence of west nile virus revisited eight decades after its first isolation. Virulence 12 (1), 1145–1173. 10.1080/21505594.2021.1908740 33843445 PMC8043182

[B142] Sarratea M. B. Alberti A. S. Redolfi D. M. Truant S. N. Iannantuono Lopez L. V. Bivona A. E. (2023). Zika virus NS4B protein targets TANK-Binding kinase 1 and inhibits type I interferon production. Biochim. Biophys. Acta Gen. Subj. 1867 (12), 130483. 10.1016/j.bbagen.2023.130483 37802371

[B143] Schoggins J. W. (2019). Interferon-stimulated genes: what do they all do? Annu. Rev. Virol. 6 (1), 567–584. 10.1146/annurev-virology-092818-015756 31283436

[B144] Selisko B. Wang C. Harris E. Canard B. (2014). Regulation of flavivirus RNA synthesis and replication. Curr. Opin. Virol. 9, 74–83. 10.1016/j.coviro.2014.09.011 25462437 PMC4295515

[B145] Shresta S. Kyle J. L. Snider H. M. Basavapatna M. Beatty P. R. Harris E. (2004). Interferon-dependent immunity is essential for resistance to primary dengue virus infection in mice, whereas T- and B-cell-dependent immunity are less critical. J. Virol. 78 (6), 2701–2710. 10.1128/jvi.78.6.2701-2710.2004 14990690 PMC353772

[B146] Singh P. Khatib M. N. Ballal S. Kaur M. Nathiya D. Sharma S. (2025). West nile virus in a changing climate: epidemiology, pathology, advances in diagnosis and treatment, vaccine designing and control strategies, emerging public health challenges - a comprehensive review. Emerg. Microbes Infect. 14 (1), 2437244. 10.1080/22221751.2024.2437244 39614679 PMC11703391

[B147] Smithburn K. C. Hughes T. P. Burke A. W. Paul J. H. (1940). A neurotropic virus isolated from the blood of a native of Uganda. Am. J. Trop. Med. s1-20 (4), 471–492. 10.4269/ajtmh.1940.s1-20.471

[B148] Sommereyns C. Paul S. Staeheli P. Michiels T. (2008). IFN-Lambda (IFN-lambda) is expressed in a tissue-dependent fashion and primarily acts on epithelial cells *in vivo* . PLoS Pathog. 4 (3), e1000017. 10.1371/journal.ppat.1000017 18369468 PMC2265414

[B149] Srivastava S. Chaudhary N. Ojha A. Guchhait P. Patel A. K. (2021). Signal transducer and activator of transcription 3 (STAT3) acts as a proviral factor for dengue virus propagation. Virus Res. 300, 198436. 10.1016/j.virusres.2021.198436 33901593

[B150] Suen W. W. Prow N. A. Hall R. A. Bielefeldt-Ohmann H. (2014). Mechanism of west nile virus neuroinvasion: a critical appraisal. Viruses 6 (7), 2796–2825. 10.3390/v6072796 25046180 PMC4113794

[B151] Sui L. Zhao Y. Wang W. Chi H. Tian T. Wu P. (2023). Flavivirus prM interacts with MDA5 and MAVS to inhibit RLR antiviral signaling. Cell Biosci. 13 (1), 9. 10.1186/s13578-023-00957-0 36639652 PMC9837762

[B152] Sun L. Wu J. Du F. Chen X. Chen Z. J. (2013). Cyclic GMP-AMP synthase is a cytosolic DNA sensor that activates the type I interferon pathway. Science 339 (6121), 786–791. 10.1126/science.1232458 23258413 PMC3863629

[B153] Suputtamongkol Y. Avirutnan P. Mairiang D. Angkasekwinai N. Niwattayakul K. Yamasmith E. (2021). Ivermectin accelerates circulating nonstructural protein 1 (NS1) clearance in adult dengue patients: a combined phase 2/3 randomized double-blinded placebo controlled trial. Clin. Infect. Dis. 72 (10), e586–e593. 10.1093/cid/ciaa1332 33462580

[B154] Tan Z. Wu J. Huang L. Wang T. Zheng Z. Zhang J. (2023). LGP2 directly interacts with flavivirus NS5 RNA-dependent RNA polymerase and downregulates its pre-elongation activities. PLoS Pathog. 19 (9), e1011620. 10.1371/journal.ppat.1011620 37656756 PMC10501626

[B155] Van Den Elsen K. Quek J. P. Luo D. (2021). Molecular insights into the flavivirus replication complex. Viruses 13 (6), 956. 10.3390/v13060956 34064113 PMC8224304

[B156] Van Der Sluis R. M. Cham L. B. Gris-Oliver A. Gammelgaard K. R. Pedersen J. G. Idorn M. (2022). TLR2 and TLR7 mediate distinct immunopathological and antiviral plasmacytoid dendritic cell responses to SARS-CoV-2 infection. Embo J. 41 (10), e109622. 10.15252/embj.2021109622 35178710 PMC9108609

[B157] Velandia-Romero M. L. Calderón-Peláez M. A. Castellanos J. E. (2016). *In vitro* infection with dengue virus induces changes in the structure and function of the mouse brain endothelium. PLoS One 11 (6), e0157786. 10.1371/journal.pone.0157786 27336851 PMC4919088

[B158] Verma S. Lo Y. Chapagain M. Lum S. Kumar M. Gurjav U. (2009). West nile virus infection modulates human brain microvascular endothelial cells tight junction proteins and cell adhesion molecules: transmigration across the *in vitro* blood-brain barrier. Virology 385 (2), 425–433. 10.1016/j.virol.2008.11.047 19135695 PMC2684466

[B159] Viettri M. Caraballo G. Sanchez M. E. Espejel-Nuñez A. Betanzos A. Ortiz-Navarrete V. (2023). Comparative infections of zika, dengue, and yellow fever viruses in human cytotrophoblast-derived cells suggest a gating role for the cytotrophoblast in zika virus placental invasion. Microbiol. Spectr. 11 (3), e0063023. 10.1128/spectrum.00630-23 37227282 PMC10269719

[B160] Walker F. C. Sridhar P. R. Baldridge M. T. (2021). Differential roles of interferons in innate responses to mucosal viral infections. Trends Immunol. 42 (11), 1009–1023. 10.1016/j.it.2021.09.003 34629295 PMC8496891

[B161] Wang K. Zou S. Chen H. Higazy D. Gao X. Zhang Y. (2023). Zika virus replication on endothelial cells and invasion into the central nervous system by inhibiting interferon β translation. Virology 582, 23–34. 10.1016/j.virol.2023.03.006 36996689

[B162] Watanabe S. Chan K. W. K. Tan N. W. W. Mahid M. B. A. Chowdhury A. Chang K. T. E. (2022). Experimental evidence for a high rate of maternal-fetal transmission of dengue virus in the presence of antibodies in immunocompromised mice. EBioMedicine 77, 103930. 10.1016/j.ebiom.2022.103930 35290828 PMC8921544

[B163] Weisblum Y. Oiknine-Djian E. Vorontsov O. M. Haimov-Kochman R. Zakay-Rones Z. Meir K. (2017). Zika virus infects Early- and midgestation human maternal decidual tissues, inducing distinct innate tissue responses in the maternal-fetal interface. J. Virol. 91 (4), e01905-16. 10.1128/JVI.01905-16 27974560 PMC5286880

[B164] Wells A. I. Coyne C. B. (2018). Type III interferons in antiviral defenses at barrier surfaces. Trends Immunol. 39 (10), 848–858. 10.1016/j.it.2018.08.008 30219309 PMC6179363

[B165] Whitehead S. S. Blaney J. E. Durbin A. P. Murphy B. R. (2007). Prospects for a dengue virus vaccine. Nat. Rev. Microbiol. 5 (7), 518–528. 10.1038/nrmicro1690 17558424

[B166] Wicherska-Pawłowska K. Wróbel T. Rybka J. (2021). Toll-like receptors (TLRs), NOD-like receptors (NLRs), and RIG-I-Like receptors (RLRs) in innate immunity. TLRs, NLRs, and RLRs ligands as immunotherapeutic agents for hematopoietic diseases. Int. J. Mol. Sci. 22 (24). 10.3390/ijms222413397 34948194 PMC8704656

[B167] Wilson J. R. De Sessions P. F. Leon M. A. Scholle F. (2008). West nile virus nonstructural protein 1 inhibits TLR3 signal transduction. J. Virol. 82 (17), 8262–8271. 10.1128/JVI.00226-08 18562533 PMC2519649

[B168] Xiang X. Yu D. Li Z. Fros J. J. Wei J. Liu K. (2024). Japanese encephalitis virus-induced DNA methylation contributes to blood-brain barrier permeability by modulating tight junction protein expression. J. Neuroinflammation 21 (1), 277. 10.1186/s12974-024-03266-6 39468601 PMC11520778

[B169] Xiong J. Yang L. Nan X. Zhu S. Yan M. Xiang S. (2025). Extracellular vesicles promote the infection and pathogenicity of Japanese encephalitis virus. J. Extracell. Vesicles 14 (1), e70033. 10.1002/jev2.70033 39783853 PMC11714208

[B170] Yang T. C. Li S. W. Lai C. C. Lu K. Z. Chiu M. T. Hsieh T. H. (2013). Proteomic analysis for type I interferon antagonism of Japanese encephalitis virus NS5 protein. Proteomics 13 (23-24), 3442–3456. 10.1002/pmic.201300001 24166946 PMC7167617

[B171] Yang Q. You J. Zhou Y. Wang Y. Pei R. Chen X. (2020). Tick-borne encephalitis virus NS4A ubiquitination antagonizes type I interferon-stimulated STAT1/2 signalling pathway. Emerg. Microbes Infect. 9 (1), 714–726. 10.1080/22221751.2020.1745094 32196427 PMC7170394

[B172] Yang C. Chen W. Huang Y. (2024). Long non-coding RNA SUN2-AS1 acts as a negative regulator of ISGs transcription to promote flavivirus infection. Virology 600, 110245. 10.1016/j.virol.2024.110245 39288611

[B173] Ye J. Chen Z. Li Y. Zhao Z. He W. Zohaib A. (2017). Japanese encephalitis virus NS5 inhibits type I interferon (IFN) production by blocking the nuclear translocation of IFN regulatory factor 3 and NF-κB. J. Virol. 91 (8). 10.1128/JVI.00039-17 28179530 PMC5375679

[B174] Ye H. Duan X. Yao M. Kang L. Li Y. Li S. (2021). USP18 mediates interferon resistance of dengue virus infection. Front. Microbiol. 12, 682380. 10.3389/fmicb.2021.682380 34017322 PMC8130619

[B175] Yin Y. Favoreel H. W. (2021). Herpesviruses and the type III interferon system. Virol. Sin. 36 (4), 577–587. 10.1007/s12250-020-00330-2 33400088 PMC8379308

[B176] Yockey L. J. Jurado K. A. Arora N. Millet A. Rakib T. Milano K. M. (2018). Type I interferons instigate fetal demise after zika virus infection. Sci. Immunol. 3 (19). 10.1126/sciimmunol.aao1680 29305462 PMC6049088

[B177] Yoneyama M. Fujita T. (2007). Function of RIG-I-like receptors in antiviral innate immunity. J. Biol. Chem. 282 (21), 15315–15318. 10.1074/jbc.R700007200 17395582

[B178] Yoneyama M. Onomoto K. Jogi M. Akaboshi T. Fujita T. (2015). Viral RNA detection by RIG-I-like receptors. Curr. Opin. Immunol. 32, 48–53. 10.1016/j.coi.2014.12.012 25594890

[B179] Zanluca C. Melo V. C. Mosimann A. L. Santos G. I. V. D. Santos C. N. D. D. Luz K. (2015). First report of autochthonous transmission of zika virus in Brazil. Mem. Inst. Oswaldo Cruz 110 (4), 569–572. 10.1590/0074-02760150192 26061233 PMC4501423

[B180] Zeng Q. Liu J. Hao C. Zhang B. Zhang H. (2023a). Making sense of flavivirus non-strctural protein 1 in innate immune evasion and inducing tissue-specific damage. Virus Res. 336, 199222. 10.1016/j.virusres.2023.199222 37716670 PMC10518729

[B181] Zeng Q. Liu J. Li Z. Zhang Y. Zu S. Ding X. (2023b). Japanese encephalitis virus NS4B inhibits interferon beta production by targeting TLR3 and TRIF. Vet. Microbiol. 284, 109849. 10.1016/j.vetmic.2023.109849 37597377

[B182] Zhang H. L. Ye H. Q. Liu S. Q. Deng C. L. Li X. D. Shi P. Y. (2017). West nile virus NS1 antagonizes interferon beta production by targeting RIG-I and MDA5. J. Virol. 91 (18). 10.1128/JVI.02396-16 28659477 PMC5571242

[B183] Zhang Y. G. Chen H. W. Zhang H. X. Wang K. Su J. Chen Y. R. (2022). EGFR activation impairs antiviral activity of interferon signaling in brain microvascular endothelial cells during Japanese encephalitis virus infection. Front. Microbiol. 13, 894356. 10.3389/fmicb.2022.894356 35847084 PMC9279666

[B184] Zhang Y. Sheng Z. Chen Q. Zhou A. Cao J. Xue F. (2023). Neutrophil infiltration leads to fetal growth restriction by impairing the placental vasculature in DENV-Infected pregnant mice. EBioMedicine 95, 104739. 10.1016/j.ebiom.2023.104739 37544202 PMC10432184

[B185] Zhang Y. G. Zhang H. X. Chen H. W. Lv P. Su J. Chen Y. R. (2023). Type I/type III IFN and related factors regulate JEV infection and BBB endothelial integrity. J. Neuroinflammation 20 (1), 216. 10.1186/s12974-023-02891-x 37752509 PMC10523659

[B186] Zhang L. Nan X. Zhou D. Wang X. Zhu S. Li Q. (2024). Japanese encephalitis virus NS1 and NS1' protein disrupts the blood-brain barrier through macrophage migration inhibitory factor-mediated autophagy. J. Virol. 98 (5), e0011624. 10.1128/jvi.00116-24 38591880 PMC11092347

[B187] Zheng Y. Li M. Wang H. Liang G. (2012). Japanese encephalitis and Japanese encephalitis virus in mainland China. Rev. Med. Virol. 22 (5), 301–322. 10.1002/rmv.1710 22407526

[B188] Zhu H. Zheng C. (2020). The race between host antiviral innate immunity and the immune evasion strategies of Herpes simplex virus 1. Microbiol. Mol. Biol. Rev. 84 (4). 10.1128/MMBR.00099-20 32998978 PMC7528619

[B189] Zoladek J. Nisole S. (2023). Mosquito-borne flaviviruses and type I interferon: catch me if you can. Front. Microbiol. 14, 1257024. 10.3389/fmicb.2023.1257024 37965539 PMC10642725

[B190] Zou S. S. Zou Q. C. Xiong W. J. Cui N. Y. Wang K. Liu H. X. (2021). Brain microvascular endothelial cell-derived HMGB1 facilitates monocyte adhesion and transmigration to promote JEV neuroinvasion. Front. Cell Infect. Microbiol. 11, 701820. 10.3389/fcimb.2021.701820 34532298 PMC8439198

